# Inflammatory pathways and immune dysregulation in pediatric postoperative septic shock: A study integrating transcriptomics, machine learning and molecular docking

**DOI:** 10.1097/MD.0000000000045205

**Published:** 2025-10-17

**Authors:** Guangshen Zhang, Lanxiang Chen, Hua Wang, Yingchao Ye, Taosheng Zhou

**Affiliations:** a Department of Surgery, Maoming Maternal and Child Health Hospital, Maoming, Guangdong Province, China; b Department of Pediatrics, Maonan District Maternal and Child Health Hospital, Maoming, Guangdong Province, China.

**Keywords:** gene expression, immune microenvironment, Mendelian randomization, molecular docking, pediatric postoperative septic shock

## Abstract

This study elucidates the molecular and immune regulatory mechanisms of pediatric postoperative septic shock. Transcriptomic data were obtained from the Gene Expression Omnibus database. Differentially expressed genes were identified using the limma package, and gene co-expression modules were constructed using Weighted Gene Co-expression Network Analysis. Functional enrichment was performed via gene set enrichment analysis, Gene Ontology, and Kyoto Encyclopedia of Genes and Genomes analyses. Immune cell infiltration was assessed using ESTIMATE and CIBERSORT. Mendelian randomization was applied to explore causal relationships between gene expression and septic shock. Feature genes were selected using machine learning algorithms, and a diagnostic nomogram model was constructed. Finally, molecular docking analysis was performed to screen and evaluate the binding affinity of traditional Chinese medicine monomers to core target proteins. A total of 1331 differentially expressed genes were identified, and the turquoise module was strongly correlated with septic shock. Enrichment analysis revealed significant activation of IL-6/JAK/STAT3, TNF-α/NF-κB, and PI3K/Akt/mTOR pathways. Immune infiltration analysis indicated suppressed immune scores and imbalances in neutrophils, macrophages, T cells, and B cells. Mendelian randomization confirmed causal associations for 6 genes, including PIM3. The predictive model based on feature genes demonstrated high diagnostic performance. Molecular docking suggested that quercetin and astramembrannin I could stably bind PIM3. This study systematically identified core genes, dysregulated immune pathways, and candidate small-molecule interventions in pediatric septic shock, providing novel insights for early diagnosis and targeted therapy.

## 1. Introduction

Pediatric postoperative septic shock is one of the most serious complications in pediatric surgery.^[1]^ The primary causes include postoperative infection, intestinal barrier disruption, gut-derived infections, systemic inflammatory response imbalance, and immune dysfunction.^[2,3]^ Due to the immature immune system in children, postoperative infections can rapidly activate systemic inflammatory response syndrome, leading to microcirculatory disorders, hypovolemic shock, and multiple organ dysfunction syndrome, which can progress to refractory septic shock.^[4,5]^ Despite advancements in intensive care, anti-infection strategies, and organ support technologies, some children still die due to the rapid progression of the condition and poor therapeutic response.^[6]^ Even if survival is achieved, some children may suffer from long-term complications such as growth retardation, neurocognitive dysfunction, and immune deficiency.^[7,8]^

Recent studies have shown that septic shock involves not only a systemic inflammatory response but also coagulation disorders, mitochondrial dysfunction, and gut microbiota dysbiosis. Overactivation of the coagulation system can lead to disseminated intravascular coagulation, worsening microcirculatory disorders, and further driving uncontrolled inflammation.^[9]^ Mitochondrial dysfunction is considered a key pathological factor in septic shock. Studies have found that metabolic disturbances, excessive reactive oxygen species release, and reduced ATP production in septic shock patients may exacerbate cell damage and organ failure.^[10–12]^ Gut microbiota plays an important role in maintaining immune homeostasis and intestinal barrier function. However, the gut microbiota structure often changes significantly in septic shock patients, with a reduction in beneficial bacteria and an increase in opportunistic pathogens, further aggravating the inflammatory response and impairing intestinal barrier function.^[13,14]^ These findings underscore that pediatric postoperative septic shock results from the complex interplay of immune, metabolic, and microbial factors. However, precise molecular biomarkers and effective targeted therapies remain lacking.

To bridge this gap, we conducted a comprehensive analysis based on transcriptomic data from pediatric septic shock patients. By integrating weighted gene co-expression network analysis (WGCNA), gene set enrichment analysis (GSEA), immune infiltration profiling, and mendelian randomization (MR), we identified key regulatory genes and potential causal pathways. Furthermore, molecular docking was employed to evaluate candidate therapeutic compounds from traditional Chinese medicine (TCM), offering novel insights into early diagnosis and intervention strategies.

## 2. Methods

### 2.1. Data acquisition and standardization

Gene expression data related to pediatric septic shock were retrieved from the Gene Expression Omnibus (GEO) database using the search terms: (“sepsis” OR “septic shock” OR “severe sepsis” OR “systemic inflammatory response syndrome” OR “SIRS”) AND “human” AND (“gene expression” OR “RNA-seq” OR “microarray”). The retrieved datasets were divided into a training set and validation sets for downstream analyses and machine learning modeling. Data preprocessing was performed in R, including conditional log₂(x + 1) transformation for non-log transformed datasets and quantile normalization across samples using the normalizeBetweenArrays function from the limma package.

### 2.2. Differential gene analysis and weighted gene co-expression network construction

Differential expression analysis was performed on the training dataset using the limma package, defining significant genes as |logFC| > 1 and adjusted *P*-value < 0.05. WGCNA was then applied with the top 25% most variable genes. An optimal softPower, the threshold parameter in WGCNA used to ensure scale-free topology, was determined. Gene modules were identified through topological overlap and hierarchical clustering, and their correlations with clinical traits were assessed and visualized by heatmaps.

### 2.3. Gene set enrichment analysis

To identify significantly altered pathways between pediatric septic shock and control groups, GSEA was performed using normalized gene expression data of training dataset. Genes were ranked based on logFC values, and enrichment analysis was conducted with the “h.all.v2024.1.Hs.symbols.gmt” gene set using the GSEA function from the clusterProfiler package. Pathways with *P*-value <  .05 were considered significant. The top 5 positively enriched pathways in the septic shock group were visualized.

### 2.4. Intersection gene screening and functional enrichment analysis

Differentially expressed genes were intersected with key modules from WGCNA to identify core genes associated with pediatric septic shock. Gene ontology (GO) and Kyoto Encyclopedia of Genes and Genomes (KEGG) enrichment analyses were performed using the clusterProfiler package, with pathways meeting adjusted *P*-value < 0.05 considered significant. Enrichment results were visualized to highlight GO terms related to inflammation and immunity, and KEGG pathways associated with disease mechanisms. To explore molecular interactions, a protein–protein interaction network was constructed using Metascape.

### 2.5. Immune cell infiltration analysis

To characterize the immune landscape of pediatric septic shock, immune infiltration analysis was performed on the training dataset. The ESTIMATE algorithm, implemented via the estimate R package, generated stromal, immune, and ESTIMATE scores, which were compared between septic shock and control groups using a *t*-test. CIBERSORT was used to quantify the relative proportions of 22 immune cell types. Only samples with *P*-value <  .05 were retained, and group differences in immune cell infiltration were assessed using the Wilcoxon rank-sum test.

### 2.6. MR analysis and nomogram construction

To investigate the genetic causal relationships underlying pediatric septic shock, MR analysis was conducted using expression quantitative trait loci data as exposures and sepsis GWAS data from the IEU Open GWAS Project (https://gwas.mrcieu.ac.uk/) as outcomes. Single nucleotide polymorphisms with *F*-statistics > 10 and no linkage disequilibrium were selected as instrumental variables. Five MR methods were applied, including the inverse variance weighted method, MR-Egger, simple mode, weighted median, and weighted mode. Genes showing causal associations with sepsis were identified based on inverse variance weighted *P*-value < .05 and pleiotropy *P*-value >  .05. Genes with odds ratio > 1 were intersected with both upregulated genes and WGCNA module genes, and forest plots were used to visualize the MR results. A logistic regression model was then built based on these intersected genes using the rms R package. A nomogram was generated to visualize individual risk prediction, and model calibration was assessed using bootstrap derived calibration curves. Decision curve analysis was also performed to evaluate the model’s clinical utility across different risk thresholds.

### 2.7. Machine learning-based feature gene selection

To identify feature genes associated with pediatric septic shock, this study integrated multiple machine learning algorithms. A total of 99 model combinations based on Lasso regression, support vector machine, random forest, gradient boosting machine, and extreme gradient boosting were used to construct classification models from standardized gene expression data of the training dataset. The area under the curve was calculated for each model to assess performance, and the optimal model was selected accordingly. Receiver operating characteristic curves were plotted to evaluate the classification performance in both training and validation sets. Differential expression of feature genes was visualized by volcano plots in the training set, and standardized mean differences in the validation set were used to verify expression consistency.

### 2.8. Functional enrichment and immune correlation analysis of feature genes

GSEA was conducted using the training dataset to investigate the biological functions of feature genes identified via machine learning. Samples of pediatric septic shock were divided into high- and low-expression groups based on feature gene expression levels, and logFC values were calculated accordingly. Enrichment analysis was performed using the “h.all.v2024.1.Hs.symbols.gmt” gene set from MSigDB. Pathways were ranked by enrichment score, and those with *P*-value < 0.05 were considered statistically significant. The top 5 enriched pathways were visualized. Additionally, to evaluate the association between feature gene expression and immune infiltration, Spearman correlation analysis was conducted by gene expression data with immune cell fractions estimated via CIBERSORT.

### 2.9. Screening and molecular docking of TCM monomers

To identify potential therapeutic compounds targeting proteins encoded by pediatric septic shock feature genes, TCM monomers were screened using the HERB database (http://herb.ac.cn/). The structures of candidate monomers and target proteins were obtained from PubChem (https://pubchem.ncbi.nlm.nih.gov/) and the AlphaFold protein structure database (https://alphafold.ebi.ac.uk/), respectively. Molecular docking was conducted via CB-Dock2 (https://cadd.labshare.cn/cb-dock2/php/blinddock.php) to evaluate the binding affinity between TCM monomers and target proteins.

## 3. Results

### 3.1. Datasets related to pediatric septic shock

**Figure 1.**
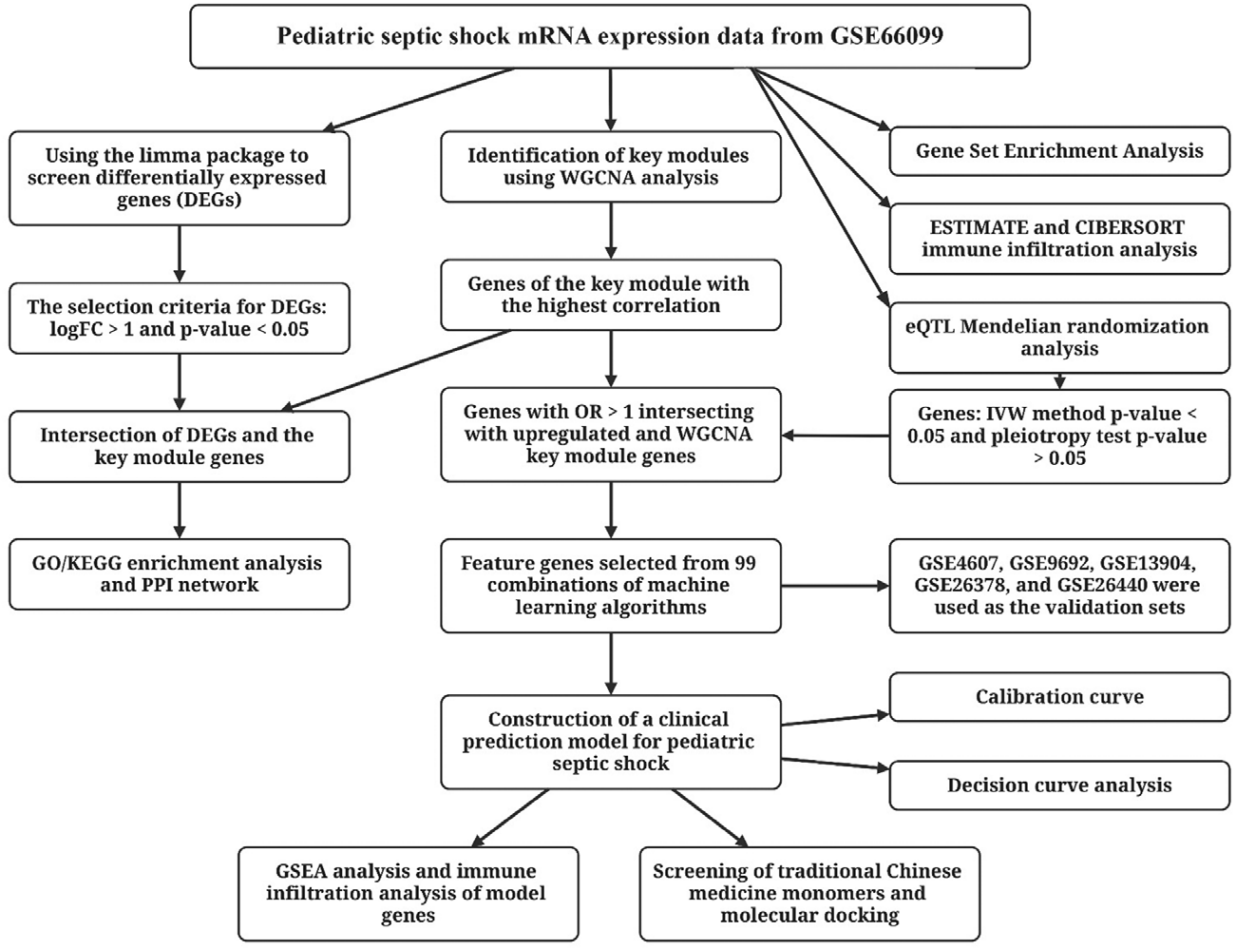
Workflow of this study. This study utilized data from the GEO database, applied the limma method for differential gene analysis, and constructed a gene co-expression network using WGCNA. The relevant pathways were explored through GSEA, GO, and KEGG analyses, while immune cell infiltration was evaluated using ESTIMATE and CIBERSORT. Mendelian randomization analysis was used to explore the causal relationships between gene expression and septic shock. Machine learning was applied to select feature genes, and a predictive model was constructed. Finally, molecular docking analysis was used to assess potential therapeutic molecules. GEO = Gene Expression Omnibus, GO = gene ontology, GSEA = gene set enrichment analysis, KEGG = Kyoto Encyclopedia of Genes and Genomes, WGCNA = weighted gene co-expression network analysis.

The study design was outlined in Figure 1. Six pediatric septic shock related datasets were selected and normalized from the GEO database. The GSE66099 dataset was used as the training set, comprising whole blood gene expression data from 181 patients and 47 healthy controls. The GSE4607, GSE9692, GSE13904, GSE26378, and GSE26440 datasets served as validation sets, including a total of 385 patients and 101 healthy controls.

### 3.2. Differentially expressed genes and core gene module

**Figure 2.**
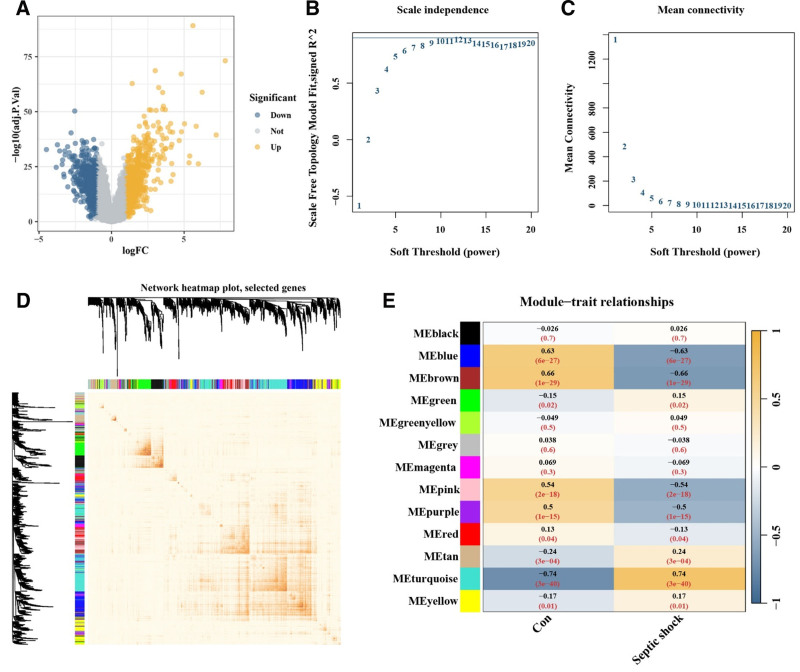
Differential expression genes and weighted gene co-expression network analysis in pediatric septic shock. (A) Volcano plot showing the 1331 significantly differentially expressed genes in the GSE66099 dataset. (B and C) Soft threshold of 9 used in WGCNA analysis. (D) Network heatmap showing the similarity and co-expression patterns between selected genes. (E) Module-phenotype correlation analysis indicating that the MEturquoise module has the strongest correlation with pediatric septic shock. WGCNA = weighted gene co-expression network analysis.

A total of 1331 differentially expressed genes were identified in pediatric septic shock samples compared to controls (Fig. 2A). Co-expression network analysis further revealed 13 gene modules, among which the turquoise module, a gene cluster labeled by turquoise color in WGCNA and most strongly correlated with the septic shock phenotype, was identified as the key module (Fig. 2B–E).

### 3.3. Key signaling pathways in pediatric septic shock

**Figure 3.**
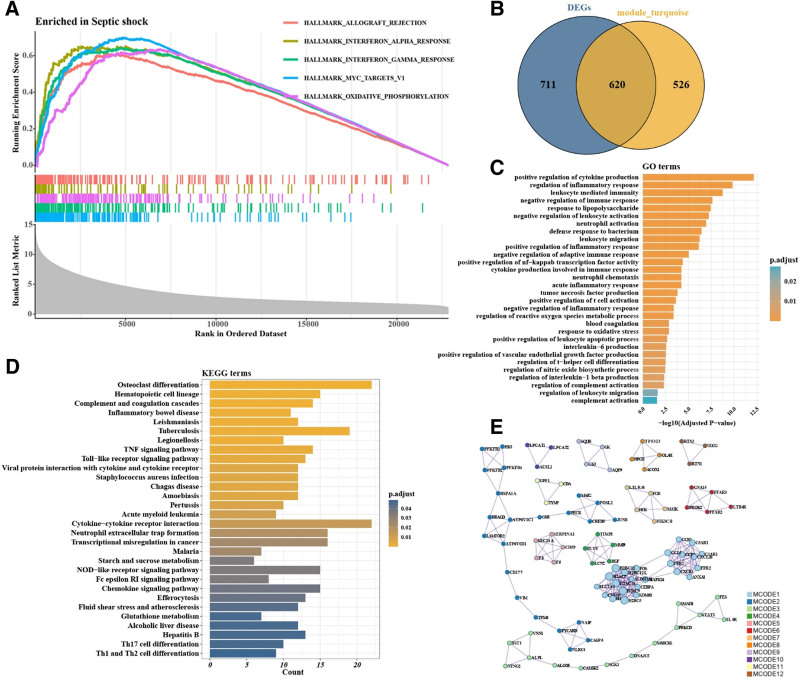
Pathway and gene function analysis in pediatric septic shock. (A) Top 5 significantly enriched pathways identified by GSEA. (B) 620 intersection genes selected through differentially expressed genes and the turquoise module. (C) The intersection genes are significantly enriched in 30 GO terms related to pediatric septic shock, involving key processes such as inflammation and immune regulation. (D) KEGG analysis identified 30 enriched pathways covering biological processes related to immunity, infection, and metabolic disorders. (E) Protein–protein interaction analysis identified 12 key functional modules primarily related to immune response, cell function, and metabolism. GO = gene ontology, GSEA = gene set enrichment analysis, KEGG = Kyoto Encyclopedia of Genes and Genomes, WGCNA = weighted gene co-expression network analysis.

GSEA revealed 33 significantly altered pathways in pediatric septic shock (Table 1). Among them, allograft rejection, interferon-α and interferon-γ responses, MYC targets, and oxidative phosphorylation were most prominently activated (Fig. 3A). These pathways are closely associated with immune activation and metabolic reprogramming, suggesting their potential involvement in the pathophysiology of pediatric septic shock.

**Table 1:** GSEA analysis identified 33 significantly enriched pathways in pediatric septic shock samples.

Description	enrichmentScore	NES	*P*.adjust
HALLMARK_MYC_TARGETS_V1	6.98E−01	2.75E+00	3.50E−10
HALLMARK_INTERFERON_GAMMA_RESPONSE	6.42E−01	2.53E+00	3.50E−10
HALLMARK_OXIDATIVE_PHOSPHORYLATION	6.34E−01	2.49E+00	3.50E−10
HALLMARK_INTERFERON_ALPHA_RESPONSE	6.50E−01	2.46E+00	3.50E−10
HALLMARK_ALLOGRAFT_REJECTION	6.08E−01	2.39E+00	3.50E−10
HALLMARK_PROTEIN_SECRETION	6.21E−01	2.35E+00	3.50E−10
HALLMARK_UNFOLDED_PROTEIN_RESPONSE	5.93E−01	2.26E+00	3.50E−10
HALLMARK_DNA_REPAIR	5.62E−01	2.18E+00	3.50E−10
HALLMARK_MTORC1_SIGNALING	5.39E−01	2.12E+00	3.50E−10
HALLMARK_HEME_METABOLISM	5.35E−01	2.10E+00	3.50E−10
HALLMARK_COMPLEMENT	5.19E−01	2.04E+00	3.50E−10
HALLMARK_ADIPOGENESIS	4.98E−01	1.96E+00	3.50E−10
HALLMARK_APOPTOSIS	4.92E−01	1.92E+00	3.50E−10
HALLMARK_TNFA_SIGNALING_VIA_NFKB	4.74E−01	1.86E+00	3.50E−10
HALLMARK_P53_PATHWAY	4.71E−01	1.85E+00	4.13E−10
HALLMARK_FATTY_ACID_METABOLISM	4.88E−01	1.90E+00	5.19E−10
HALLMARK_PI3K_AKT_MTOR_SIGNALING	5.34E−01	2.03E+00	7.78E−10
HALLMARK_INFLAMMATORY_RESPONSE	4.53E−01	1.78E+00	5.53E−09
HALLMARK_E2F_TARGETS	4.53E−01	1.78E+00	2.68E−08
HALLMARK_REACTIVE_OXYGEN_SPECIES_PATHWAY	6.20E−01	2.19E+00	2.97E−08
HALLMARK_HYPOXIA	4.43E−01	1.74E+00	6.15E−08
HALLMARK_IL2_STAT5_SIGNALING	4.40E−01	1.73E+00	1.60E−07
HALLMARK_UV_RESPONSE_UP	4.54E−01	1.77E+00	4.42E−07
HALLMARK_ANDROGEN_RESPONSE	4.82E−01	1.83E+00	5.58E−06
HALLMARK_IL6_JAK_STAT3_SIGNALING	4.71E−01	1.77E+00	2.11E−05
HALLMARK_G2M_CHECKPOINT	4.08E−01	1.60E+00	2.71E−05
HALLMARK_MITOTIC_SPINDLE	3.73E−01	1.47E+00	7.40E−04
HALLMARK_TGF_BETA_SIGNALING	4.77E−01	1.71E+00	1.73E−03
HALLMARK_PEROXISOME	4.07E−01	1.55E+00	2.91E−03
HALLMARK_GLYCOLYSIS	3.47E−01	1.37E+00	1.17E−02
HALLMARK_KRAS_SIGNALING_UP	3.44E−01	1.35E+00	1.22E−02
HALLMARK_MYC_TARGETS_V2	4.13E−01	1.49E+00	2.37E−02
HALLMARK_CHOLESTEROL_HOMEOSTASIS	3.88E−01	1.43E+00	3.81E−02

### 3.4. Functional characterization of key genes associated with pediatric septic shock

A total of 620 intersecting genes were identified between the differentially expressed genes and the turquoise module (Fig. 3B). GO enrichment analysis indicated significant involvement in 531 pathways across molecular function, biological process, and cellular component categories (Table S1, Supplemental Digital Content, https://links.lww.com/MD/Q356). Among these, 30 GO terms were closely associated with pediatric septic shock, including inflammatory response, immune regulation, coagulation dysfunction, and oxidative stress (Fig. 3C). KEGG analysis further revealed 30 enriched pathways related to immune response, infection, metabolic disorders, and inflammation (Fig. 3D). Protein–protein interaction network identified 12 functional modules, predominantly associated with immune and inflammatory regulation, cellular metabolism, signal transduction, and fibrosis (Fig. 3E, Table 2).

**Table 2:** PPI analysis via the Metascape database and the MCODE algorithm identified 12 key functional modules with biological functions.

MCODE	GO	Description	Log10(P)
MCODE_1	hsa04613	Neutrophil extracellular trap formation	−17.9
MCODE_1	GO:0006935	Chemotaxis	−17.1
MCODE_1	GO:0042330	Taxis	−17.1
MCODE_2	GO:0046835	Carbohydrate phosphorylation	−8.7
MCODE_2	GO:0006003	Fructose 2,6-bisphosphate metabolic process	−8.5
MCODE_2	hsa00051	Fructose and mannose metabolism	−8
MCODE_3	WP5094	Orexin receptor pathway	−9.4
MCODE_3	R-HSA-163125	Posttranslational modification: synthesis of GPI-anchored proteins	−7.2
MCODE_3	GO:0016310	Phosphorylation	−6
MCODE_4	R-HSA-6785807	Interleukin-4 and Interleukin-13 signaling	−9.5
MCODE_4	WP3624	Lung fibrosis	−7.1
MCODE_4	M167	PID AP1 PATHWAY	−6.9
MCODE_5	R-HSA-5694530	Cargo concentration in the ER	−11.5
MCODE_5	R-HSA-204005	COPII-mediated vesicle transport	−10.2
MCODE_5	hsa04610	Complement and coagulation cascades	−9.5
MCODE_6	R-HSA-416476	G alpha (q) signaling events	−11
MCODE_6	R-HSA-388396	GPCR downstream signaling	−8.6
MCODE_6	R-HSA-372790	Signaling by GPCR	−8.4
MCODE_7	M141	PID PI3KCI PATHWAY	−7.4
MCODE_7	M124	PID CXCR4 PATHWAY	−6.5
MCODE_7	M186	PID PDGFRB PATHWAY	−6.1
MCODE_8	GO:0006631	Fatty acid metabolic process	−5.3
MCODE_8	GO:0032787	Monocarboxylic acid metabolic process	−4.7
MCODE_8	R-HSA-556833	Metabolism of lipids	−4.3
MCODE_10	GO:0045017	Glycerolipid biosynthetic process	−6.5
MCODE_10	GO:0046486	Glycerolipid metabolic process	−5.8
MCODE_10	GO:0090407	Organophosphate biosynthetic process	−5.2
MCODE_11	R-HSA-73614	Pyrimidine salvage	−10.6
MCODE_11	GO:0006213	Pyrimidine nucleoside metabolic process	−9.8
MCODE_11	R-HSA-8956321	Nucleotide salvage	−9.5
MCODE_12	GO:0010256	Endomembrane system organization	−5.2

### 3.5. Immune microenvironment characteristics and immune cell abundance in pediatric septic shock

**Figure 4.**
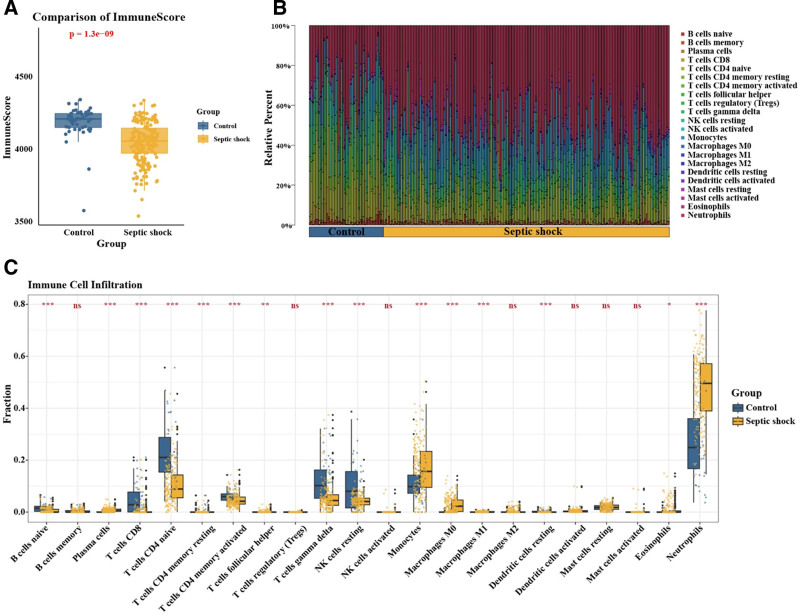
Immune score and immune cell infiltration analysis in pediatric septic shock. (A) ESTIMATE analysis shows a significantly lower immune score in the pediatric septic shock group compared to the control group, suggesting that septic shock may be associated with immune suppression. (B) CIBERSORT analysis shows differences in the relative abundance of 22 immune cell types between the pediatric septic shock and control groups. (C) Boxplot of immune cell infiltration analysis showing significant differences in the relative abundance of 22 immune cell types between the pediatric septic shock group and the control group.

ESTIMATE analysis indicated a significantly reduced immune score in pediatric septic shock patients compared to controls, suggesting a state of immune suppression (Fig. 4A). CIBERSORT analysis further revealed notable alterations in immune cell composition (Fig. 4B). The septic shock samples exhibited increased infiltration of plasma cells, resting memory CD4⁺ T cells, follicular helper T cells, monocytes, M0 and M1 macrophages, eosinophils, and neutrophils. Conversely, the proportions of naive B cells, CD8⁺ T cells, naive and activated memory CD4⁺ T cells, γδ T cells, resting NK cells, and resting dendritic cells were significantly decreased (Fig. 4C).

### 3.6. MR analysis results and predictive model

**Figure 5.**
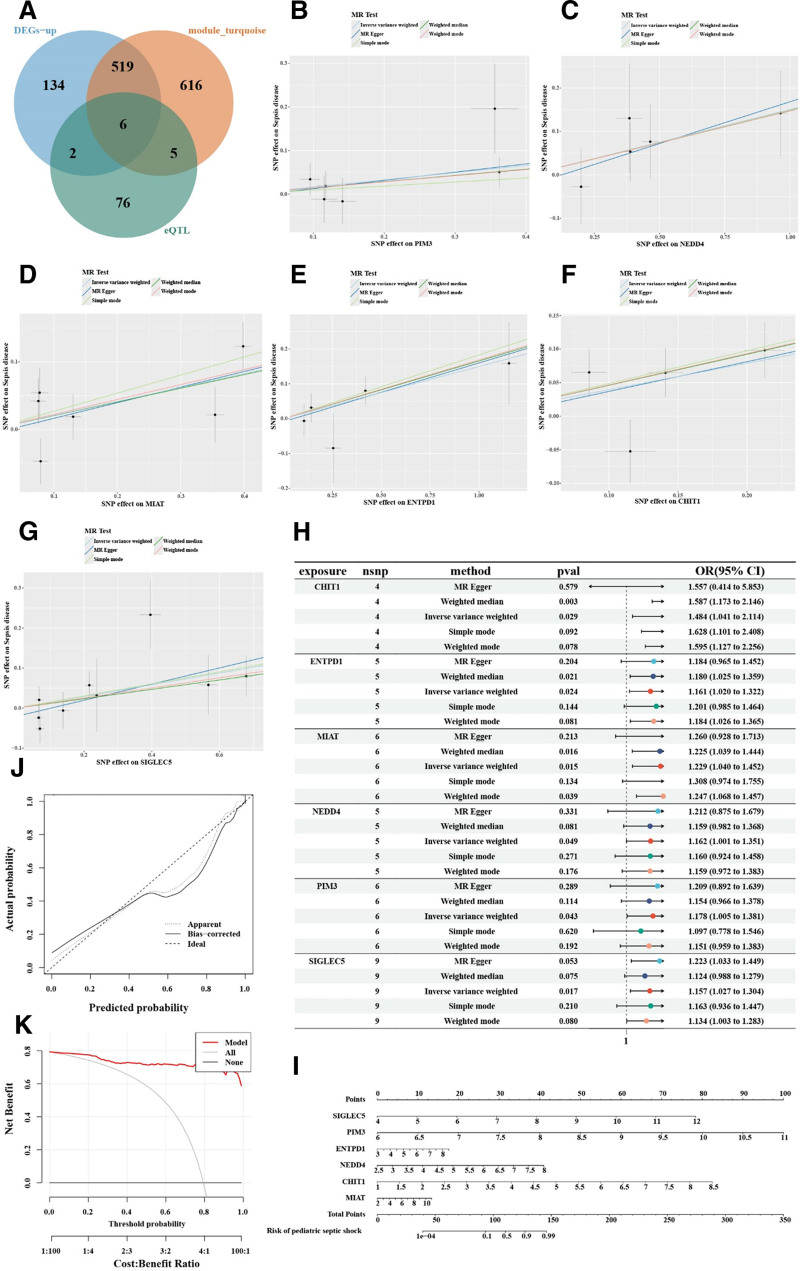
Mendelian randomization analysis and predictive model construction for pediatric septic shock. (A) Differential expression gene analysis, WGCNA analysis, and eQTL Mendelian randomization analysis identified 6 core genes significantly associated with septic shock outcomes. (B–G) Results of Mendelian randomization analysis showing that upregulation of core gene expression is significantly associated with poor outcomes in sepsis. (H) Risk ratios and 95% confidence intervals for the 6 core genes under different analysis methods. (I) A nomogram-based risk prediction model for pediatric septic shock constructed using 6 core genes. (J) Calibration curve showing the high reliability of the model in terms of prediction accuracy. (K) Decision curve analysis showing that the model has good clinical utility at different thresholds and can effectively distinguish between pediatric septic shock patients and healthy controls. WGCNA = weighted gene co-expression network analysis. eQTL = expression quantitative trait loci.

MR analysis based on expression quantitative trait loci data identified 179 genes significantly associated with sepsis outcomes (ieu-b-5086), including 89 genes with odds ratios > 1 and 90 with odds ratios < 1. SIGLEC5, PIM3, ENTPD1, NEDD4, CHIT1, and MIAT were selected by intersecting upregulated genes with those showing causal associations (Fig. 5A). Elevated expression of these genes was significantly linked to adverse sepsis outcomes (Fig. 5B–H). A nomogram was constructed based on these core genes (Fig. 5I), and calibration curves demonstrated good predictive performance (Fig. 5J). Decision curve analysis further confirmed the model’s clinical utility across multiple threshold probabilities, effectively distinguishing pediatric septic shock patients from healthy controls (Fig. 5K).

### 3.7. Feature genes identified by machine learning

**Figure 6.**
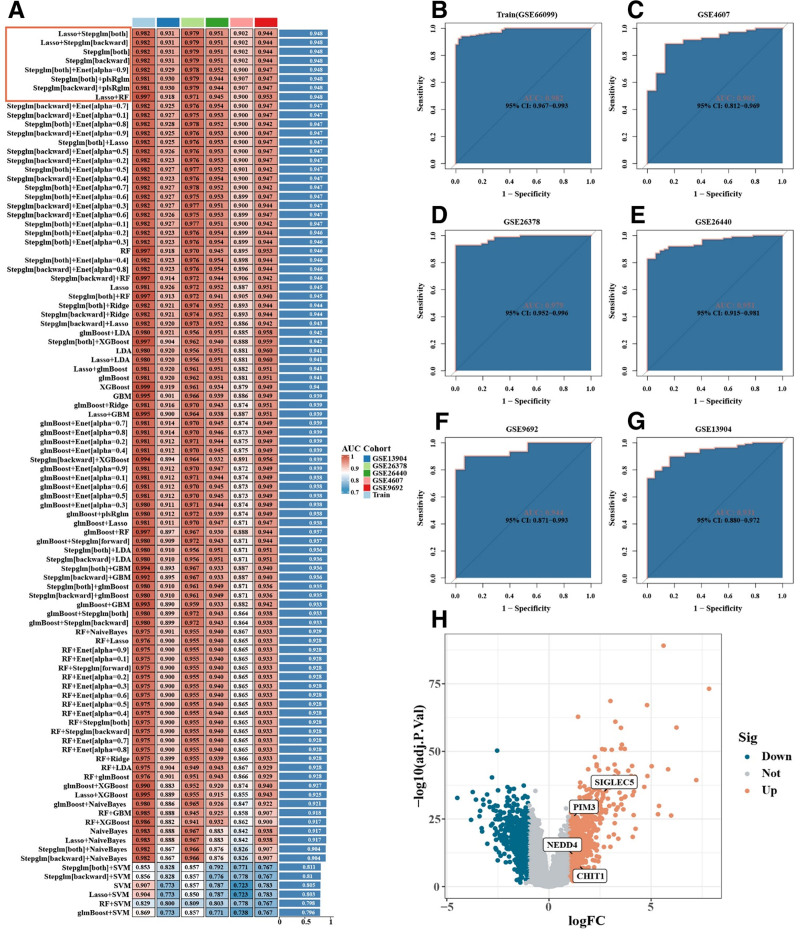
Machine learning feature gene selection and classification performance evaluation in pediatric septic shock. (A) Among 99 algorithm combinations, 8 combinations showed the best area under the curve values. (B–G) The feature genes (PIM3, SIGLEC5, CHIT1, and NEDD4) selected based on optimal algorithm combinations showed good classification performance in both training and validation sets. Receiver operating characteristic curves demonstrate the classification efficiency of feature genes in different datasets. (H) Volcano plot analysis shows significant upregulation of selected feature genes in the training set.

**Figure 7.**
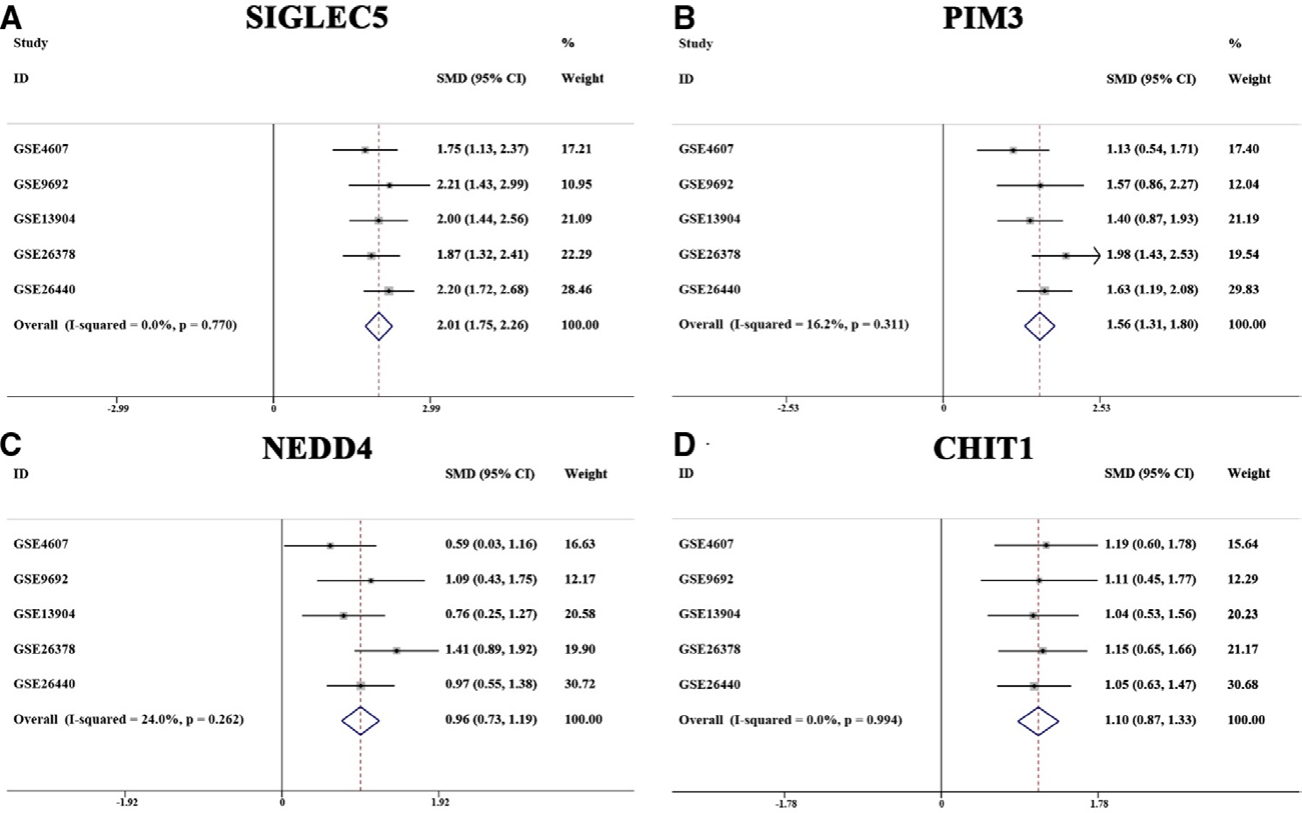
Expression analysis of feature genes in the validation set for pediatric septic shock. (A–D) In the validation set, the expression of PIM3, SIGLEC5, CHIT1, and NEDD4 was significantly upregulated in pediatric septic shock patients.

To identify robust biomarkers of pediatric septic shock, multiple machine learning algorithms were applied. Among 99 model combinations, 8 demonstrated optimal classification performance, with an average area under the curve of 0.948 (Fig. 6A). The 4 feature genes were PIM3, SIGLEC5, CHIT1 and NEDD4, all of which demonstrated strong discriminative ability between patients and controls (Fig. 6B–G). These genes were significantly upregulated in the training set (Fig. 6H), and the upregulation trend was consistently validated in independent datasets (Fig. 7A–D).

### 3.8. Functional implications of feature genes

**Figure 8.**
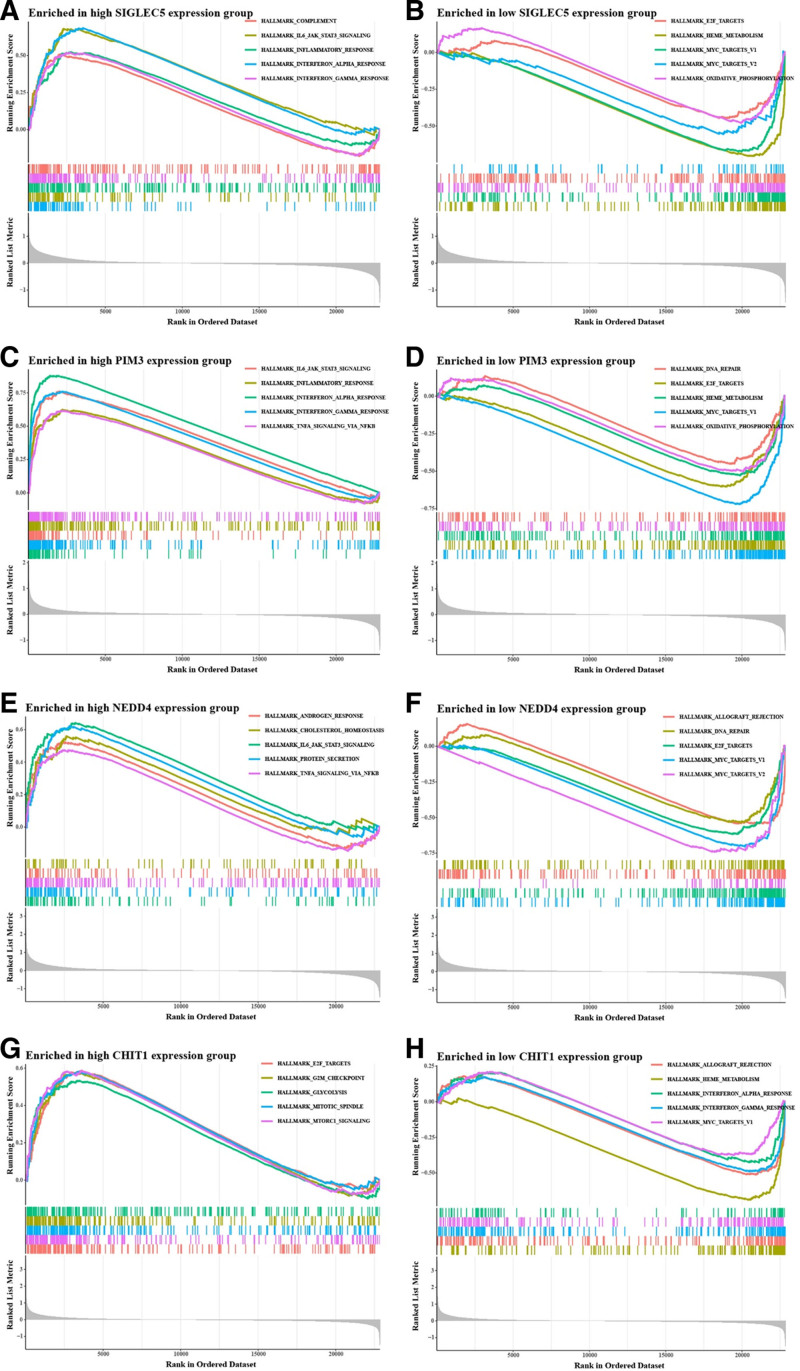
GSEA enrichment analysis of feature genes in pediatric septic shock. (A–B) In the SIGLEC5 high-expression group, complement activation, IL-6/JAK/STAT3, and interferon signaling pathways were significantly enriched, while metabolic and cell cycle-related pathways were suppressed. (C and D) In the PIM3 high-expression group, inflammation and TNF-α/NF-κB signaling pathways were activated, while DNA repair and metabolic pathways were suppressed. (E and F) In the NEDD4 high-expression group, cholesterol homeostasis, IL-6/JAK/STAT3, and TNF-α/NF-κB signaling pathways were enriched, while DNA repair and cell cycle-related pathways were suppressed. (G and H) In the CHIT1 high-expression group, glycolysis and mTORC1 pathways were activated, while transplant rejection and interferon signaling pathways were suppressed.

GSEA showed that the 4 feature genes were strongly associated with dysregulated inflammatory signaling and metabolic imbalance in pediatric septic shock. SIGLEC5 was linked to complement activation, IL-6/JAK/STAT3, and PI3K/Akt/mTOR pathways (Fig. 8A and B, Table 3). PIM3 was enriched in IL-6/JAK/STAT3, TNF-α/NF-κB, and interferon signaling (Fig. 8C and D, Table 4). NEDD4 was associated with cholesterol homeostasis, IL-6/JAK/STAT3, and TNF-α/NF-κB pathways (Fig. 8E and F, Table 5). CHIT1 was related to glycolysis, PI3K/Akt/mTOR, and mTORC1 activation (Fig. 8G and H, Table 6). These results suggest that the feature genes may jointly drive excessive inflammation and disrupt metabolic homeostasis, thereby promoting disease progression.

**Table 3:** GSEA analysis results for SIGLEC5 high-expression pediatric septic shock patients.

Description	enrichmentScore	NES	p.adjust	qvalue
HALLMARK_HEME_METABOLISM	−7.05E−01	−2.51E+00	2.50E−09	1.37E−09
HALLMARK_MYC_TARGETS_V1	−6.74E−01	−2.38E+00	2.50E−09	1.37E−09
HALLMARK_INTERFERON_ALPHA_RESPONSE	6.86E−01	2.13E+00	1.34E−07	7.32E−08
HALLMARK_IL6_JAK_STAT3_SIGNALING	6.82E−01	2.10E+00	2.72E−07	1.49E−07
HALLMARK_INFLAMMATORY_RESPONSE	5.25E−01	1.77E+00	2.08E−05	1.14E−05
HALLMARK_INTERFERON_GAMMA_RESPONSE	5.20E−01	1.75E+00	5.55E−05	3.04E−05
HALLMARK_OXIDATIVE_PHOSPHORYLATION	−4.80E−01	−1.70E+00	7.03E−05	3.85E−05
HALLMARK_COMPLEMENT	5.08E−01	1.71E+00	1.16E−04	6.33E−05
HALLMARK_MITOTIC_SPINDLE	4.98E−01	1.68E+00	1.38E−04	7.55E−05
HALLMARK_COAGULATION	5.34E−01	1.73E+00	1.89E−04	1.04E−04
HALLMARK_TNFA_SIGNALING_VIA_NFKB	4.85E−01	1.63E+00	5.26E−04	2.88E−04
HALLMARK_E2F_TARGETS	−4.47E−01	−1.59E+00	5.77E−04	3.16E−04
HALLMARK_APICAL_JUNCTION	4.84E−01	1.63E+00	7.41E−04	4.06E−04
HALLMARK_HYPOXIA	4.72E−01	1.58E+00	7.41E−04	4.06E−04
HALLMARK_MYC_TARGETS_V2	−5.56E−01	−1.67E+00	1.03E−02	5.63E−03
HALLMARK_CHOLESTEROL_HOMEOSTASIS	5.02E−01	1.50E+00	3.34E−02	1.83E−02
HALLMARK_ALLOGRAFT_REJECTION	−3.80E−01	−1.35E+00	3.58E−02	1.96E−02
HALLMARK_DNA_REPAIR	−4.00E−01	−1.39E+00	4.87E−02	2.67E−02
HALLMARK_APOPTOSIS	4.10E−01	1.35E+00	4.87E−02	2.67E−02
HALLMARK_PI3K_AKT_MTOR_SIGNALING	4.51E−01	1.41E+00	5.27E−02	2.88E−02
HALLMARK_P53_PATHWAY	3.91E−01	1.31E+00	5.81E−02	3.18E−02
HALLMARK_TGF_BETA_SIGNALING	5.09E−01	1.45E+00	6.93E−02	3.79E−02
HALLMARK_GLYCOLYSIS	3.87E−01	1.30E+00	7.81E−02	4.27E−02
HALLMARK_IL2_STAT5_SIGNALING	3.81E−01	1.28E+00	8.42E−02	4.61E−02

**Table 4:** GSEA analysis results for PIM3 high-expression pediatric septic shock patients.

Description	enrichmentScore	NES	p.adjust	qvalue
HALLMARK_INTERFERON_ALPHA_RESPONSE	8.79E−01	2.79E+00	7.14E−10	3.46E−10
HALLMARK_INTERFERON_GAMMA_RESPONSE	7.59E−01	2.67E+00	7.14E−10	3.46E−10
HALLMARK_MYC_TARGETS_V1	−7.19E−01	−2.55E+00	7.14E−10	3.46E−10
HALLMARK_IL6_JAK_STAT3_SIGNALING	7.61E−01	2.41E+00	7.14E−10	3.46E−10
HALLMARK_INFLAMMATORY_RESPONSE	6.25E−01	2.20E+00	7.14E−10	3.46E−10
HALLMARK_TNFA_SIGNALING_VIA_NFKB	6.15E−01	2.16E+00	7.14E−10	3.46E−10
HALLMARK_E2F_TARGETS	−6.03E−01	−2.14E+00	7.14E−10	3.46E−10
HALLMARK_HEME_METABOLISM	−5.29E−01	−1.88E+00	7.71E−07	3.73E−07
HALLMARK_COMPLEMENT	5.27E−01	1.85E+00	4.48E−06	2.17E−06
HALLMARK_OXIDATIVE_PHOSPHORYLATION	−5.03E−01	−1.78E+00	2.72E−05	1.32E−05
HALLMARK_IL2_STAT5_SIGNALING	4.52E−01	1.58E+00	2.07E−03	1.00E−03
HALLMARK_HYPOXIA	4.48E−01	1.57E+00	2.32E−03	1.12E−03
HALLMARK_DNA_REPAIR	−4.51E−01	−1.55E+00	2.76E−03	1.34E−03
HALLMARK_APOPTOSIS	4.57E−01	1.56E+00	3.53E−03	1.71E−03
HALLMARK_CHOLESTEROL_HOMEOSTASIS	5.62E−01	1.73E+00	3.72E−03	1.80E−03
HALLMARK_G2M_CHECKPOINT	−4.22E−01	−1.50E+00	3.72E−03	1.80E−03
HALLMARK_MYC_TARGETS_V2	−5.77E−01	−1.72E+00	3.98E−03	1.93E−03
HALLMARK_COAGULATION	4.61E−01	1.54E+00	9.39E−03	4.54E−03
HALLMARK_PROTEIN_SECRETION	4.70E−01	1.50E+00	2.19E−02	1.06E−02
HALLMARK_MITOTIC_SPINDLE	3.89E−01	1.37E+00	4.00E−02	1.94E−02
HALLMARK_UV_RESPONSE_UP	3.96E−01	1.35E+00	4.58E−02	2.22E−02
HALLMARK_FATTY_ACID_METABOLISM	−3.80E−01	−1.31E+00	4.58E−02	2.22E−02
HALLMARK_PI3K_AKT_MTOR_SIGNALING	4.32E−01	1.39E+00	7.51E−02	3.64E−02
HALLMARK_APICAL_JUNCTION	3.75E−01	1.31E+00	7.51E−02	3.64E−02
HALLMARK_ANGIOGENESIS	−5.45E−01	−1.47E+00	8.40E−02	4.07E−02
HALLMARK_MTORC1_SIGNALING	3.68E−01	1.29E+00	8.40E−02	4.07E−02
HALLMARK_P53_PATHWAY	3.68E−01	1.29E+00	8.40E−02	4.07E−02

**Table 5:** GSEA analysis results for NEDD4 high-expression pediatric septic shock patients.

Description	enrichmentScore	NES	p.adjust	qvalue
HALLMARK_MYC_TARGETS_V1	−7.04E−01	−2.36E+00	2.50E−09	1.47E−09
HALLMARK_E2F_TARGETS	−6.17E−01	−2.07E+00	2.50E−09	1.47E−09
HALLMARK_MYC_TARGETS_V2	−7.42E−01	−2.11E+00	1.87E−06	1.10E−06
HALLMARK_ALLOGRAFT_REJECTION	−5.43E−01	−1.82E+00	3.62E−06	2.14E−06
HALLMARK_IL6_JAK_STAT3_SIGNALING	6.42E−01	1.91E+00	5.79E−05	3.41E−05
HALLMARK_PROTEIN_SECRETION	6.24E−01	1.87E+00	1.19E−04	7.03E−05
HALLMARK_DNA_REPAIR	−5.36E−01	−1.73E+00	1.77E−04	1.04E−04
HALLMARK_G2M_CHECKPOINT	−4.79E−01	−1.60E+00	6.38E−04	3.76E−04
HALLMARK_TNFA_SIGNALING_VIA_NFKB	4.74E−01	1.56E+00	1.54E−03	9.05E−04
HALLMARK_INTERFERON_GAMMA_RESPONSE	−4.60E−01	−1.55E+00	1.54E−03	9.05E−04
HALLMARK_ANDROGEN_RESPONSE	5.23E−01	1.57E+00	8.97E−03	5.29E−03
HALLMARK_CHOLESTEROL_HOMEOSTASIS	5.61E−01	1.62E+00	9.56E−03	5.63E−03
HALLMARK_INFLAMMATORY_RESPONSE	4.44E−01	1.46E+00	9.56E−03	5.63E−03
HALLMARK_HYPOXIA	4.35E−01	1.43E+00	1.65E−02	9.73E−03
HALLMARK_INTERFERON_ALPHA_RESPONSE	−4.99E−01	−1.53E+00	1.82E−02	1.07E−02
HALLMARK_OXIDATIVE_PHOSPHORYLATION	−4.08E−01	−1.36E+00	2.48E−02	1.46E−02
HALLMARK_COAGULATION	4.52E−01	1.42E+00	3.06E−02	1.80E−02
HALLMARK_ADIPOGENESIS	4.17E−01	1.37E+00	3.07E−02	1.81E−02
HALLMARK_IL2_STAT5_SIGNALING	4.03E−01	1.32E+00	5.17E−02	3.04E−02
HALLMARK_COMPLEMENT	4.07E−01	1.34E+00	6.15E−02	3.63E−02
HALLMARK_XENOBIOTIC_METABOLISM	4.06E−01	1.34E+00	6.15E−02	3.63E−02
HALLMARK_UNFOLDED_PROTEIN_RESPONSE	−4.37E−01	−1.36E+00	8.17E−02	4.81E−02

**Table 6:** GSEA analysis results for CHIT1 high-expression pediatric septic shock patients.

Description	enrichmentScore	NES	p.adjust	qvalue
HALLMARK_HEME_METABOLISM	−6.89E−01	−2.51E+00	5.00E−09	2.21E−09
HALLMARK_E2F_TARGETS	5.87E−01	1.93E+00	2.60E−06	1.15E−06
HALLMARK_ALLOGRAFT_REJECTION	−5.12E−01	−1.87E+00	2.60E−06	1.15E−06
HALLMARK_MTORC1_SIGNALING	5.85E−01	1.92E+00	2.61E−06	1.15E−06
HALLMARK_MITOTIC_SPINDLE	5.81E−01	1.92E+00	4.69E−06	2.08E−06
HALLMARK_G2M_CHECKPOINT	5.74E−01	1.88E+00	1.78E−05	7.89E−06
HALLMARK_INTERFERON_GAMMA_RESPONSE	−4.90E−01	−1.79E+00	1.78E−05	7.89E−06
HALLMARK_GLYCOLYSIS	5.33E−01	1.76E+00	2.42E−04	1.07E−04
HALLMARK_ESTROGEN_RESPONSE_LATE	5.25E−01	1.73E+00	4.50E−04	1.99E−04
HALLMARK_COAGULATION	5.47E−01	1.72E+00	7.75E−04	3.43E−04
HALLMARK_HYPOXIA	5.16E−01	1.70E+00	7.75E−04	3.43E−04
HALLMARK_CHOLESTEROL_HOMEOSTASIS	6.43E−01	1.85E+00	1.14E−03	5.05E−04
HALLMARK_IL6_JAK_STAT3_SIGNALING	6.06E−01	1.80E+00	1.40E−03	6.19E−04
HALLMARK_PROTEIN_SECRETION	5.72E−01	1.71E+00	3.16E−03	1.40E−03
HALLMARK_UNFOLDED_PROTEIN_RESPONSE	5.48E−01	1.67E+00	3.16E−03	1.40E−03
HALLMARK_ADIPOGENESIS	4.73E−01	1.55E+00	5.49E−03	2.43E−03
HALLMARK_TNFA_SIGNALING_VIA_NFKB	4.64E−01	1.53E+00	9.01E−03	3.98E−03
HALLMARK_COMPLEMENT	4.55E−01	1.50E+00	1.08E−02	4.76E−03
HALLMARK_ANDROGEN_RESPONSE	5.29E−01	1.59E+00	1.23E−02	5.43E−03
HALLMARK_SPERMATOGENESIS	4.75E−01	1.49E+00	2.73E−02	1.21E−02
HALLMARK_MYC_TARGETS_V1	−3.76E−01	−1.37E+00	2.73E−02	1.21E−02
HALLMARK_INTERFERON_ALPHA_RESPONSE	−4.28E−01	−1.41E+00	3.27E−02	1.44E−02
HALLMARK_INFLAMMATORY_RESPONSE	4.17E−01	1.38E+00	3.90E−02	1.72E−02
HALLMARK_IL2_STAT5_SIGNALING	4.18E−01	1.37E+00	3.90E−02	1.72E−02
HALLMARK_EPITHELIAL_MESENCHYMAL_TRANSITION	4.24E−01	1.40E+00	4.51E−02	2.00E−02
HALLMARK_REACTIVE_OXYGEN_SPECIES_PATHWAY	5.49E−01	1.48E+00	7.96E−02	3.52E−02
HALLMARK_PI3K_AKT_MTOR_SIGNALING	4.64E−01	1.41E+00	7.96E−02	3.52E−02
HALLMARK_APICAL_JUNCTION	4.01E−01	1.31E+00	7.96E−02	3.52E−02
HALLMARK_XENOBIOTIC_METABOLISM	3.96E−01	1.30E+00	7.96E−02	3.52E−02

### 3.9. Associations between feature genes and immune infiltration

**Figure 9.**
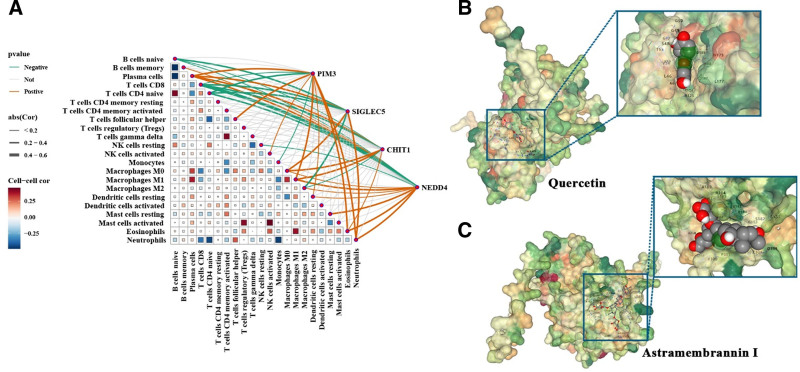
Correlation between feature genes and immune cell infiltration, and screening of traditional Chinese medicine candidate molecules. (A) Immune cell infiltration analysis shows that the expression of core genes such as SIGLEC5, PIM3, NEDD4, and CHIT1 is significantly associated with the infiltration levels of various immune cell types. (B and C) Molecular docking analysis shows that quercetin and astramembrannin I have strong binding affinity with PIM3, with AutoDock Vina scores of −7.9 and −8.7.

Correlation analysis demonstrated that the feature genes were closely linked to immune cell alterations (Fig. 9A). SIGLEC5 was positively correlated with M0 macrophages and neutrophils but negatively with CD8⁺ T cells and M2 macrophages, reflecting a pro-inflammatory yet immunosuppressive shift. PIM3 was associated with increased M1 macrophages and neutrophils but reduced CD8⁺ T cells, indicating enhanced inflammatory polarization. NEDD4 correlated with plasma cells, macrophage subsets, and neutrophils, while CHIT1 was linked to M0/M1 macrophages and eosinophils, both accompanied by decreased cytotoxic cell populations. These patterns highlight that the feature genes may not only activate inflammatory pathways but also reshape the immune microenvironment, underscoring their potential as biomarkers and therapeutic targets.

### 3.10. Herbal candidate molecules and molecular docking evaluation

Candidate therapeutic compounds associated with the feature genes were identified using the HERB database. Based on literature support, quercetin and astramembrannin I (also known as astragaloside A) were selected for further evaluation. Quercetin, a widely distributed flavonoid, has been reported to inhibit the NF-κB and PI3K/AKT/mTOR pathways, thereby exerting anti-inflammatory effects.^[15,16]^ Astramembrannin I, a principal active component of *Astragalus membranaceus*, exhibits antioxidant, anti-apoptotic, and anti-inflammatory activities.^[17]^ Molecular docking analysis demonstrated stable binding of both compounds to PIM3, with AutoDock vina binding energies of −7.9 kcal/mol for quercetin and −8.7 kcal/mol for astramembrannin I (Fig. 9B and C).

## 4. Discussion

This study integrated transcriptomic analysis, immune infiltration profiling, MR analysis, and molecular docking to explore the mechanisms of pediatric septic shock. The IL-6/JAK/STAT3, PI3K/Akt/mTOR, and TNF-α/NF-κB pathways were identified as central drivers, accompanied by marked alterations in neutrophils, macrophages, T cells, and B cells. Causal associations between core genes and clinical outcomes were established through MR analysis, and a nomogram based on these genes demonstrated strong diagnostic performance. Molecular docking further indicated that quercetin and astramembrannin I may target PIM3, suggesting potential therapeutic value.

At the molecular level, the progression of pediatric septic shock involves dysregulation of multiple inflammatory signaling pathways. IL-6 activates the JAK/STAT3 pathway, promoting the synthesis of acute phase proteins and regulating immune cell activation and proliferation.^[18]^ The PI3K/Akt/mTOR pathway plays a critical role in cellular metabolism and immune regulation.^[19]^ Its sustained activation may induce immune exhaustion and organ dysfunction.^[20]^ TNF-α/NF-κB signaling enhances inflammatory responses by inducing pro-inflammatory cytokines and activating immune cells, but its overactivation may trigger cytokine storms and immune imbalance, ultimately leading to multi-organ failure in septic shock.^[21–23]^ These pathways interact to form a complex inflammatory regulatory network that underlies immune dysregulation in pediatric septic shock.

Immune infiltration analysis further highlighted the roles of specific immune cell subsets in the pathogenesis of pediatric septic shock. Macrophages can polarize into pro-inflammatory M1 or anti-inflammatory M2 phenotypes depending on microenvironmental cues. M1 macrophages enhance inflammatory responses and host defense through the secretion of cytokines such as TNF-α, IL-6, and IL-12, but their excessive activation may lead to tissue damage.^[24,25]^ In contrast, M2 macrophages secrete IL-10 and TGF-β, promoting tissue repair and dampening inflammation.^[24]^ Our analysis revealed a significant increase in M1 macrophages in pediatric septic shock patients, indicating an intensified inflammatory response and compromised tissue repair capacity, which may contribute to organ dysfunction. Notably, the mTORC1 pathway is a central signaling cascade regulating cellular metabolism and immune responses.^[26]^ Its activation promotes M1 polarization, whereas its inhibition facilitates M2 polarization.^[27]^ In our study, CHIT1 overexpression was closely associated with enrichment of the mTORC1 signaling pathway, indicating that CHIT1 may drive M1 polarization and inhibit M2 differentiation through mTORC1 activation, thereby intensifying inflammation and impairing tissue repair. In addition to macrophages, other immune cell subsets also exhibited significant alterations in pediatric septic shock. Neutrophils were markedly elevated, while CD8⁺ T cells, NK cells, and B cells were notably decreased. Neutrophils play a critical role in the early phase of sepsis by phagocytosing and eliminating pathogens.^[28]^ However, their excessive activation can lead to the release of reactive oxygen species, neutrophil extracellular traps, and proteases, which damage the endothelial barrier and trigger systemic inflammation.^[29]^ In this study, elevated expression of SIGLEC5, PIM3, and NEDD4 was positively correlated with neutrophil infiltration, suggesting that these genes may facilitate neutrophil recruitment by regulating cytokines and chemokines. Moreover, excessive neutrophils can suppress adaptive immunity by inhibiting T cell activation and proliferation.^[30]^ CD8⁺ T cells are essential for pathogen clearance, and their depletion may impair cytotoxic responses, increasing the risk of secondary infections in the later stages of sepsis.^[31,32]^ Further analysis showed that high expression of SIGLEC5, PIM3, CHIT1, and NEDD4 was significantly negatively correlated with CD8⁺ T cell infiltration, indicating their potential role in promoting immunosuppression in pediatric septic shock. NK cells contribute to host defense by recognizing and eliminating infected cells and modulating immune responses through cytokine secretion.^[33]^ A retrospective study reported that lower NK cell levels were associated with higher 28-day mortality in sepsis patients.^[34]^ Furthermore, B cell depletion may impair antibody-mediated immunity and weaken long-term protection against pathogens.^[35]^ Taken together, immune dysregulation in pediatric septic shock is characterized by excessive activation of pro-inflammatory cells and depletion of key immune effector populations. Core genes such as PIM3, NEDD4, CHIT1, and SIGLEC5 appear to play regulatory roles in shaping immune cell infiltration and may serve as promising targets for therapeutic intervention.

Based on the identified immune and pathway features, this study further screened potential therapeutic compounds. Quercetin, a flavonoid compound, has well-established anti-inflammatory and antioxidant effects.^[36]^ Studies have shown that quercetin can alleviate LPS-induced myocardial injury by modulating the ALOX5/PI3K/AKT pathway and promote macrophage M2 polarization while reducing IL-6 levels via suppression of the PI3K/Akt/mTOR and JAK2/STAT3 pathways.^[16,37,38]^ Additionally, quercetin inhibits NF-κB and AP1 signaling, thereby reducing TNF-α-induced apoptosis and inflammatory responses in human umbilical vein endothelial cells.^[15]^ Our molecular docking analysis revealed that quercetin binds stably to PIM3, suggesting that it may exert anti-inflammatory effects through this target via multiple signaling pathways. Astramembrannin I, a principal component of Astragalus membranaceus, also exhibits antioxidant, anti-inflammatory, and anti-apoptotic properties.^[17]^ Docking results indicated that both compounds showed strong binding affinities to PIM3, supporting their potential as therapeutic candidates.

In summary, this study revealed the critical involvement of the IL-6/JAK/STAT3, PI3K/Akt/mTOR, and TNF-α/NF-κB pathways in pediatric postoperative septic shock and identified key genes such as PIM3, with quercetin and astramembrannin I predicted as potential therapeutic compounds. While these findings provide novel mechanistic insights, several limitations must be emphasized. The molecular docking results are hypothesis-generating only, as binding affinity predictions cannot confirm therapeutic efficacy. The absence of functional validation remains a major gap. Future studies should therefore focus on qPCR or immunohistochemistry validation of candidate genes in patient samples, in vitro functional assays, and in vivo evaluation of therapeutic compounds in animal models. Such efforts, together with single-cell sequencing and validation in independent clinical cohorts, will be essential to strengthen the robustness and translational relevance of our findings.

## 5. Conclusion

This study identified IL-6/JAK/STAT3, TNF-α/NF-κB, and PI3K/Akt/mTOR pathways as central to pediatric septic shock and highlighted PIM3 as a potential therapeutic target. Quercetin and astramembrannin I demonstrated binding affinity to PIM3, suggesting therapeutic relevance. These findings offer insights into pathogenesis and inform targeted intervention strategies.

## Acknowledgments

We thank the Department of Surgery at Maoming Maternal and Child Health Hospital for their support, as well as the GEO database, IEU Open GWAS Project database, and CB-Dock2 platform for providing the data and tools.

## Author contributions

**Data curation:** Taosheng Zhou.

**Investigation:** Guangshen Zhang, Taosheng Zhou.

**Methodology:** Guangshen Zhang, Taosheng Zhou.

**Project administration:** Lanxiang Chen.

**Resources:** Yingchao Ye.

**Software:** Lanxiang Chen, Yingchao Ye.

**Visualization:** Hua Wang.

**Writing – original draft:** Hua Wang, Yingchao Ye, Taosheng Zhou.

**Writing – review & editing:** Taosheng Zhou.

## Supplementary Material


