# Supplementary material for: Inflammatory pathways and immune dysregulation in pediatric postoperative septic shock: A study integrating transcriptomics, machine learning and molecular docking
Source: Medicine (Baltimore). 2025 Oct 17;104(42):e45205. doi: 10.1097/MD.0000000000045205 (PMC12537127; doi:10.1097/MD.0000000000045205)
Supplement: Supplementary file 1 [file medi-104-e45205-s001.docx]

**Supplementary Table S1.** GO enrichment analysis results of 620 intersecting genes between differentially expressed genes and the turquoise module.

| ONTOLOGY | ID | Description | pvalue | p.adjust | qvalue |
| --- | --- | --- | --- | --- | --- |
| BP | GO:0002366 | leukocyte activation involved in immune response | 3.07E-17 | 1.04E-13 | 8.45E-14 |
| BP | GO:0002263 | cell activation involved in immune response | 4.95E-17 | 1.04E-13 | 8.45E-14 |
| BP | GO:0002274 | myeloid leukocyte activation | 1.43E-16 | 2.01E-13 | 1.62E-13 |
| BP | GO:0002697 | regulation of immune effector process | 4.65E-16 | 4.90E-13 | 3.97E-13 |
| BP | GO:0001819 | positive regulation of cytokine production | 8.32E-16 | 7.02E-13 | 5.68E-13 |
| BP | GO:0050727 | regulation of inflammatory response | 2.42E-13 | 1.48E-10 | 1.20E-10 |
| BP | GO:0031349 | positive regulation of defense response | 2.45E-13 | 1.48E-10 | 1.20E-10 |
| BP | GO:0002764 | immune response-regulating signaling pathway | 1.05E-12 | 5.56E-10 | 4.50E-10 |
| BP | GO:0002703 | regulation of leukocyte mediated immunity | 2.83E-12 | 1.33E-09 | 1.07E-09 |
| BP | GO:0002286 | T cell activation involved in immune response | 3.14E-12 | 1.33E-09 | 1.07E-09 |
| BP | GO:0002698 | negative regulation of immune effector process | 4.40E-12 | 1.69E-09 | 1.37E-09 |
| BP | GO:0001906 | cell killing | 4.85E-12 | 1.71E-09 | 1.38E-09 |
| BP | GO:0002443 | leukocyte mediated immunity | 5.43E-12 | 1.76E-09 | 1.43E-09 |
| BP | GO:0002460 | adaptive immune response based on somatic recombination of immune receptors built from immunoglobulin superfamily domains | 8.97E-12 | 2.71E-09 | 2.19E-09 |
| BP | GO:0045088 | regulation of innate immune response | 1.67E-11 | 4.71E-09 | 3.81E-09 |
| BP | GO:0002819 | regulation of adaptive immune response | 2.79E-11 | 7.35E-09 | 5.95E-09 |
| BP | GO:0002768 | immune response-regulating cell surface receptor signaling pathway | 4.43E-11 | 1.10E-08 | 8.90E-09 |
| BP | GO:0002285 | lymphocyte activation involved in immune response | 4.86E-11 | 1.14E-08 | 9.22E-09 |
| BP | GO:0050777 | negative regulation of immune response | 1.10E-10 | 2.36E-08 | 1.91E-08 |
| BP | GO:0002699 | positive regulation of immune effector process | 1.13E-10 | 2.36E-08 | 1.91E-08 |
| BP | GO:0002757 | immune response-activating signaling pathway | 1.21E-10 | 2.36E-08 | 1.91E-08 |
| BP | GO:0002292 | T cell differentiation involved in immune response | 1.23E-10 | 2.36E-08 | 1.91E-08 |
| BP | GO:0050866 | negative regulation of cell activation | 1.40E-10 | 2.58E-08 | 2.08E-08 |
| BP | GO:0031341 | regulation of cell killing | 2.12E-10 | 3.64E-08 | 2.94E-08 |
| BP | GO:0032496 | response to lipopolysaccharide | 2.15E-10 | 3.64E-08 | 2.94E-08 |
| BP | GO:0002449 | lymphocyte mediated immunity | 2.42E-10 | 3.94E-08 | 3.19E-08 |
| BP | GO:0002237 | response to molecule of bacterial origin | 2.61E-10 | 4.08E-08 | 3.30E-08 |
| BP | GO:0006935 | chemotaxis | 3.13E-10 | 4.72E-08 | 3.82E-08 |
| BP | GO:0042330 | taxis | 3.55E-10 | 5.17E-08 | 4.18E-08 |
| BP | GO:0002695 | negative regulation of leukocyte activation | 4.26E-10 | 6.00E-08 | 4.86E-08 |
| BP | GO:0002706 | regulation of lymphocyte mediated immunity | 5.04E-10 | 6.86E-08 | 5.55E-08 |
| BP | GO:0036230 | granulocyte activation | 5.30E-10 | 6.99E-08 | 5.66E-08 |
| BP | GO:0006909 | phagocytosis | 5.60E-10 | 7.16E-08 | 5.79E-08 |
| BP | GO:0002822 | regulation of adaptive immune response based on somatic recombination of immune receptors built from immunoglobulin superfamily domains | 8.67E-10 | 1.08E-07 | 8.71E-08 |
| BP | GO:0042119 | neutrophil activation | 9.61E-10 | 1.16E-07 | 9.38E-08 |
| BP | GO:0002833 | positive regulation of response to biotic stimulus | 1.01E-09 | 1.19E-07 | 9.62E-08 |
| BP | GO:0050764 | regulation of phagocytosis | 2.12E-09 | 2.40E-07 | 1.94E-07 |
| BP | GO:0007159 | leukocyte cell-cell adhesion | 2.16E-09 | 2.40E-07 | 1.94E-07 |
| BP | GO:0045089 | positive regulation of innate immune response | 2.34E-09 | 2.53E-07 | 2.05E-07 |
| BP | GO:0060326 | cell chemotaxis | 2.46E-09 | 2.59E-07 | 2.10E-07 |
| BP | GO:0042742 | defense response to bacterium | 3.55E-09 | 3.65E-07 | 2.96E-07 |
| BP | GO:0042088 | T-helper 1 type immune response | 5.20E-09 | 5.23E-07 | 4.23E-07 |
| BP | GO:0050867 | positive regulation of cell activation | 6.22E-09 | 5.97E-07 | 4.83E-07 |
| BP | GO:0050900 | leukocyte migration | 6.22E-09 | 5.97E-07 | 4.83E-07 |
| BP | GO:0002825 | regulation of T-helper 1 type immune response | 6.57E-09 | 6.16E-07 | 4.99E-07 |
| BP | GO:0050729 | positive regulation of inflammatory response | 8.36E-09 | 7.67E-07 | 6.20E-07 |
| BP | GO:0042116 | macrophage activation | 9.53E-09 | 8.56E-07 | 6.93E-07 |
| BP | GO:0051250 | negative regulation of lymphocyte activation | 1.00E-08 | 8.80E-07 | 7.12E-07 |
| BP | GO:0001909 | leukocyte mediated cytotoxicity | 1.18E-08 | 1.01E-06 | 8.21E-07 |
| BP | GO:0030595 | leukocyte chemotaxis | 1.53E-08 | 1.30E-06 | 1.05E-06 |
| BP | GO:0002715 | regulation of natural killer cell mediated immunity | 1.76E-08 | 1.45E-06 | 1.18E-06 |
| BP | GO:0002429 | immune response-activating cell surface receptor signaling pathway | 2.21E-08 | 1.80E-06 | 1.45E-06 |
| BP | GO:0002696 | positive regulation of leukocyte activation | 2.78E-08 | 2.21E-06 | 1.79E-06 |
| BP | GO:0050868 | negative regulation of T cell activation | 2.99E-08 | 2.33E-06 | 1.89E-06 |
| BP | GO:0032609 | type II interferon production | 3.20E-08 | 2.36E-06 | 1.91E-06 |
| BP | GO:0032649 | regulation of type II interferon production | 3.20E-08 | 2.36E-06 | 1.91E-06 |
| BP | GO:0035710 | CD4-positive, alpha-beta T cell activation | 3.20E-08 | 2.36E-06 | 1.91E-06 |
| BP | GO:0002228 | natural killer cell mediated immunity | 3.24E-08 | 2.36E-06 | 1.91E-06 |
| BP | GO:0002705 | positive regulation of leukocyte mediated immunity | 4.12E-08 | 2.95E-06 | 2.38E-06 |
| BP | GO:0030100 | regulation of endocytosis | 4.76E-08 | 3.35E-06 | 2.71E-06 |
| BP | GO:0001910 | regulation of leukocyte mediated cytotoxicity | 6.10E-08 | 4.22E-06 | 3.41E-06 |
| BP | GO:0042269 | regulation of natural killer cell mediated cytotoxicity | 7.64E-08 | 5.07E-06 | 4.11E-06 |
| BP | GO:0032102 | negative regulation of response to external stimulus | 7.69E-08 | 5.07E-06 | 4.11E-06 |
| BP | GO:1903131 | mononuclear cell differentiation | 7.69E-08 | 5.07E-06 | 4.11E-06 |
| BP | GO:0002275 | myeloid cell activation involved in immune response | 8.09E-08 | 5.25E-06 | 4.25E-06 |
| BP | GO:0071222 | cellular response to lipopolysaccharide | 8.90E-08 | 5.69E-06 | 4.60E-06 |
| BP | GO:1903037 | regulation of leukocyte cell-cell adhesion | 1.06E-07 | 6.67E-06 | 5.40E-06 |
| BP | GO:0002708 | positive regulation of lymphocyte mediated immunity | 1.07E-07 | 6.67E-06 | 5.40E-06 |
| BP | GO:0022407 | regulation of cell-cell adhesion | 1.42E-07 | 8.67E-06 | 7.01E-06 |
| BP | GO:0002287 | alpha-beta T cell activation involved in immune response | 1.55E-07 | 9.11E-06 | 7.37E-06 |
| BP | GO:0002293 | alpha-beta T cell differentiation involved in immune response | 1.55E-07 | 9.11E-06 | 7.37E-06 |
| BP | GO:0042267 | natural killer cell mediated cytotoxicity | 1.55E-07 | 9.11E-06 | 7.37E-06 |
| BP | GO:0046631 | alpha-beta T cell activation | 1.62E-07 | 9.34E-06 | 7.56E-06 |
| BP | GO:0002820 | negative regulation of adaptive immune response | 1.64E-07 | 9.34E-06 | 7.56E-06 |
| BP | GO:1903707 | negative regulation of hemopoiesis | 1.70E-07 | 9.59E-06 | 7.76E-06 |
| BP | GO:0002758 | innate immune response-activating signaling pathway | 2.04E-07 | 1.13E-05 | 9.17E-06 |
| BP | GO:1903038 | negative regulation of leukocyte cell-cell adhesion | 2.37E-07 | 1.29E-05 | 1.04E-05 |
| BP | GO:0002218 | activation of innate immune response | 2.37E-07 | 1.29E-05 | 1.04E-05 |
| BP | GO:0071219 | cellular response to molecule of bacterial origin | 2.46E-07 | 1.31E-05 | 1.06E-05 |
| BP | GO:0002827 | positive regulation of T-helper 1 type immune response | 2.98E-07 | 1.57E-05 | 1.27E-05 |
| BP | GO:0097529 | myeloid leukocyte migration | 3.07E-07 | 1.60E-05 | 1.30E-05 |
| BP | GO:0050863 | regulation of T cell activation | 3.27E-07 | 1.68E-05 | 1.36E-05 |
| BP | GO:2000515 | negative regulation of CD4-positive, alpha-beta T cell activation | 3.40E-07 | 1.73E-05 | 1.40E-05 |
| BP | GO:0031348 | negative regulation of defense response | 3.50E-07 | 1.75E-05 | 1.41E-05 |
| BP | GO:0097530 | granulocyte migration | 3.52E-07 | 1.75E-05 | 1.41E-05 |
| BP | GO:0071216 | cellular response to biotic stimulus | 4.15E-07 | 2.04E-05 | 1.65E-05 |
| BP | GO:1902106 | negative regulation of leukocyte differentiation | 4.32E-07 | 2.10E-05 | 1.70E-05 |
| BP | GO:0030217 | T cell differentiation | 5.06E-07 | 2.43E-05 | 1.96E-05 |
| BP | GO:0046636 | negative regulation of alpha-beta T cell activation | 5.15E-07 | 2.44E-05 | 1.97E-05 |
| BP | GO:0052548 | regulation of endopeptidase activity | 5.30E-07 | 2.49E-05 | 2.01E-05 |
| BP | GO:1990266 | neutrophil migration | 5.89E-07 | 2.73E-05 | 2.21E-05 |
| BP | GO:0050766 | positive regulation of phagocytosis | 6.26E-07 | 2.87E-05 | 2.32E-05 |
| BP | GO:0071621 | granulocyte chemotaxis | 6.54E-07 | 2.97E-05 | 2.40E-05 |
| BP | GO:0052547 | regulation of peptidase activity | 6.89E-07 | 3.09E-05 | 2.50E-05 |
| BP | GO:0042093 | T-helper cell differentiation | 7.30E-07 | 3.25E-05 | 2.63E-05 |
| BP | GO:0051092 | positive regulation of NF-kappaB transcription factor activity | 9.58E-07 | 4.21E-05 | 3.41E-05 |
| BP | GO:0002294 | CD4-positive, alpha-beta T cell differentiation involved in immune response | 9.87E-07 | 4.29E-05 | 3.47E-05 |
| BP | GO:0032757 | positive regulation of interleukin-8 production | 1.14E-06 | 4.90E-05 | 3.96E-05 |
| BP | GO:0002718 | regulation of cytokine production involved in immune response | 1.20E-06 | 5.08E-05 | 4.11E-05 |
| BP | GO:0022408 | negative regulation of cell-cell adhesion | 1.20E-06 | 5.08E-05 | 4.11E-05 |
| BP | GO:0030098 | lymphocyte differentiation | 1.32E-06 | 5.53E-05 | 4.47E-05 |
| BP | GO:0006968 | cellular defense response | 1.39E-06 | 5.74E-05 | 4.65E-05 |
| BP | GO:0071674 | mononuclear cell migration | 1.40E-06 | 5.74E-05 | 4.65E-05 |
| BP | GO:0002367 | cytokine production involved in immune response | 1.48E-06 | 6.01E-05 | 4.86E-05 |
| BP | GO:0045807 | positive regulation of endocytosis | 1.52E-06 | 6.06E-05 | 4.90E-05 |
| BP | GO:2000514 | regulation of CD4-positive, alpha-beta T cell activation | 1.52E-06 | 6.06E-05 | 4.90E-05 |
| BP | GO:0002526 | acute inflammatory response | 1.56E-06 | 6.08E-05 | 4.92E-05 |
| BP | GO:0030593 | neutrophil chemotaxis | 1.56E-06 | 6.08E-05 | 4.92E-05 |
| BP | GO:0071706 | tumor necrosis factor superfamily cytokine production | 1.66E-06 | 6.36E-05 | 5.14E-05 |
| BP | GO:1903555 | regulation of tumor necrosis factor superfamily cytokine production | 1.66E-06 | 6.36E-05 | 5.14E-05 |
| BP | GO:0043367 | CD4-positive, alpha-beta T cell differentiation | 1.72E-06 | 6.53E-05 | 5.29E-05 |
| BP | GO:0046632 | alpha-beta T cell differentiation | 1.82E-06 | 6.87E-05 | 5.56E-05 |
| BP | GO:0043371 | negative regulation of CD4-positive, alpha-beta T cell differentiation | 2.04E-06 | 7.54E-05 | 6.11E-05 |
| BP | GO:0045063 | T-helper 1 cell differentiation | 2.04E-06 | 7.54E-05 | 6.11E-05 |
| BP | GO:0043299 | leukocyte degranulation | 2.30E-06 | 8.43E-05 | 6.82E-05 |
| BP | GO:0010951 | negative regulation of endopeptidase activity | 2.73E-06 | 9.92E-05 | 8.03E-05 |
| BP | GO:1903039 | positive regulation of leukocyte cell-cell adhesion | 2.84E-06 | 0.000103 | 8.30E-05 |
| BP | GO:0002717 | positive regulation of natural killer cell mediated immunity | 3.22E-06 | 0.000115 | 9.32E-05 |
| BP | GO:0032612 | interleukin-1 production | 3.32E-06 | 0.000117 | 9.44E-05 |
| BP | GO:0032652 | regulation of interleukin-1 production | 3.32E-06 | 0.000117 | 9.44E-05 |
| BP | GO:0061900 | glial cell activation | 3.35E-06 | 0.000117 | 9.44E-05 |
| BP | GO:0022409 | positive regulation of cell-cell adhesion | 4.03E-06 | 0.00014 | 0.000113 |
| BP | GO:0032640 | tumor necrosis factor production | 4.59E-06 | 0.000156 | 0.000126 |
| BP | GO:0032680 | regulation of tumor necrosis factor production | 4.59E-06 | 0.000156 | 0.000126 |
| BP | GO:0002823 | negative regulation of adaptive immune response based on somatic recombination of immune receptors built from immunoglobulin superfamily domains | 4.63E-06 | 0.000156 | 0.000127 |
| BP | GO:0051091 | positive regulation of DNA-binding transcription factor activity | 4.90E-06 | 0.000164 | 0.000133 |
| BP | GO:0045620 | negative regulation of lymphocyte differentiation | 5.42E-06 | 0.00018 | 0.000146 |
| BP | GO:0032677 | regulation of interleukin-8 production | 5.50E-06 | 0.000181 | 0.000147 |
| BP | GO:0032637 | interleukin-8 production | 6.13E-06 | 0.000199 | 0.000161 |
| BP | GO:1903706 | regulation of hemopoiesis | 6.13E-06 | 0.000199 | 0.000161 |
| BP | GO:0051251 | positive regulation of lymphocyte activation | 6.21E-06 | 0.0002 | 0.000162 |
| BP | GO:0010466 | negative regulation of peptidase activity | 6.37E-06 | 0.000204 | 0.000165 |
| BP | GO:0002700 | regulation of production of molecular mediator of immune response | 6.66E-06 | 0.000211 | 0.000171 |
| BP | GO:0001774 | microglial cell activation | 7.00E-06 | 0.000221 | 0.000178 |
| BP | GO:0050870 | positive regulation of T cell activation | 7.61E-06 | 0.000237 | 0.000192 |
| BP | GO:0031343 | positive regulation of cell killing | 7.64E-06 | 0.000237 | 0.000192 |
| BP | GO:0032729 | positive regulation of type II interferon production | 8.69E-06 | 0.000267 | 0.000216 |
| BP | GO:0046639 | negative regulation of alpha-beta T cell differentiation | 8.72E-06 | 0.000267 | 0.000216 |
| BP | GO:0002283 | neutrophil activation involved in immune response | 8.88E-06 | 0.000268 | 0.000217 |
| BP | GO:0045623 | negative regulation of T-helper cell differentiation | 8.88E-06 | 0.000268 | 0.000217 |
| BP | GO:0051090 | regulation of DNA-binding transcription factor activity | 1.03E-05 | 0.00031 | 0.000251 |
| BP | GO:0030099 | myeloid cell differentiation | 1.10E-05 | 0.000327 | 0.000265 |
| BP | GO:0031342 | negative regulation of cell killing | 1.12E-05 | 0.000329 | 0.000266 |
| BP | GO:0045954 | positive regulation of natural killer cell mediated cytotoxicity | 1.12E-05 | 0.000329 | 0.000266 |
| BP | GO:0051346 | negative regulation of hydrolase activity | 1.15E-05 | 0.000335 | 0.000271 |
| BP | GO:0002269 | leukocyte activation involved in inflammatory response | 1.16E-05 | 0.000337 | 0.000272 |
| BP | GO:1902105 | regulation of leukocyte differentiation | 1.18E-05 | 0.00034 | 0.000275 |
| BP | GO:0043086 | negative regulation of catalytic activity | 1.30E-05 | 0.000371 | 0.0003 |
| BP | GO:0002720 | positive regulation of cytokine production involved in immune response | 1.43E-05 | 0.000402 | 0.000325 |
| BP | GO:0002456 | T cell mediated immunity | 1.43E-05 | 0.000402 | 0.000325 |
| BP | GO:0050728 | negative regulation of inflammatory response | 1.44E-05 | 0.000403 | 0.000326 |
| BP | GO:2000377 | regulation of reactive oxygen species metabolic process | 1.50E-05 | 0.000418 | 0.000338 |
| BP | GO:0150076 | neuroinflammatory response | 1.61E-05 | 0.000443 | 0.000359 |
| BP | GO:0006887 | exocytosis | 1.62E-05 | 0.000445 | 0.00036 |
| BP | GO:0042092 | type 2 immune response | 1.72E-05 | 0.000469 | 0.000379 |
| BP | GO:0002430 | complement receptor mediated signaling pathway | 1.73E-05 | 0.000469 | 0.00038 |
| BP | GO:0001912 | positive regulation of leukocyte mediated cytotoxicity | 1.96E-05 | 0.000522 | 0.000423 |
| BP | GO:0002704 | negative regulation of leukocyte mediated immunity | 1.96E-05 | 0.000522 | 0.000423 |
| BP | GO:0046634 | regulation of alpha-beta T cell activation | 2.04E-05 | 0.000541 | 0.000438 |
| BP | GO:0014002 | astrocyte development | 2.06E-05 | 0.000545 | 0.000441 |
| BP | GO:0002702 | positive regulation of production of molecular mediator of immune response | 2.61E-05 | 0.000684 | 0.000553 |
| BP | GO:0038094 | Fc-gamma receptor signaling pathway | 2.79E-05 | 0.000727 | 0.000588 |
| BP | GO:0002707 | negative regulation of lymphocyte mediated immunity | 2.91E-05 | 0.000753 | 0.000609 |
| BP | GO:0043434 | response to peptide hormone | 2.94E-05 | 0.000754 | 0.00061 |
| BP | GO:0042098 | T cell proliferation | 2.95E-05 | 0.000754 | 0.00061 |
| BP | GO:0072593 | reactive oxygen species metabolic process | 3.11E-05 | 0.00079 | 0.00064 |
| BP | GO:0002221 | pattern recognition receptor signaling pathway | 3.49E-05 | 0.000883 | 0.000715 |
| BP | GO:0070555 | response to interleukin-1 | 3.61E-05 | 0.000907 | 0.000734 |
| BP | GO:0030316 | osteoclast differentiation | 3.81E-05 | 0.000953 | 0.000771 |
| BP | GO:1901136 | carbohydrate derivative catabolic process | 3.84E-05 | 0.000954 | 0.000772 |
| BP | GO:0002220 | innate immune response activating cell surface receptor signaling pathway | 4.34E-05 | 0.001071 | 0.000867 |
| BP | GO:0034113 | heterotypic cell-cell adhesion | 4.40E-05 | 0.001073 | 0.000868 |
| BP | GO:0046456 | icosanoid biosynthetic process | 4.40E-05 | 0.001073 | 0.000868 |
| BP | GO:0006690 | icosanoid metabolic process | 4.52E-05 | 0.001097 | 0.000888 |
| BP | GO:0006953 | acute-phase response | 4.76E-05 | 0.001147 | 0.000928 |
| BP | GO:0070661 | leukocyte proliferation | 4.82E-05 | 0.001156 | 0.000935 |
| BP | GO:0042129 | regulation of T cell proliferation | 5.03E-05 | 0.0012 | 0.000971 |
| BP | GO:0002444 | myeloid leukocyte mediated immunity | 5.52E-05 | 0.001308 | 0.001058 |
| BP | GO:0010721 | negative regulation of cell development | 5.63E-05 | 0.001328 | 0.001075 |
| BP | GO:0007596 | blood coagulation | 5.72E-05 | 0.001341 | 0.001085 |
| BP | GO:0046209 | nitric oxide metabolic process | 5.79E-05 | 0.001345 | 0.001088 |
| BP | GO:0006979 | response to oxidative stress | 5.80E-05 | 0.001345 | 0.001088 |
| BP | GO:0071347 | cellular response to interleukin-1 | 6.03E-05 | 0.001391 | 0.001126 |
| BP | GO:0001911 | negative regulation of leukocyte mediated cytotoxicity | 6.39E-05 | 0.001458 | 0.00118 |
| BP | GO:0002726 | positive regulation of T cell cytokine production | 6.39E-05 | 0.001458 | 0.00118 |
| BP | GO:2001057 | reactive nitrogen species metabolic process | 6.47E-05 | 0.001467 | 0.001187 |
| BP | GO:0007162 | negative regulation of cell adhesion | 6.55E-05 | 0.001479 | 0.001197 |
| BP | GO:0002821 | positive regulation of adaptive immune response | 6.81E-05 | 0.001529 | 0.001238 |
| BP | GO:0001818 | negative regulation of cytokine production | 6.89E-05 | 0.001539 | 0.001246 |
| BP | GO:0032943 | mononuclear cell proliferation | 7.20E-05 | 0.0016 | 0.001294 |
| BP | GO:0045581 | negative regulation of T cell differentiation | 7.46E-05 | 0.001649 | 0.001334 |
| BP | GO:0050817 | coagulation | 7.61E-05 | 0.001673 | 0.001354 |
| BP | GO:0007599 | hemostasis | 8.51E-05 | 0.001861 | 0.001506 |
| BP | GO:0002709 | regulation of T cell mediated immunity | 9.47E-05 | 0.002061 | 0.001668 |
| BP | GO:2000108 | positive regulation of leukocyte apoptotic process | 9.97E-05 | 0.002159 | 0.001747 |
| BP | GO:0009595 | detection of biotic stimulus | 0.000103 | 0.002217 | 0.001794 |
| BP | GO:2000379 | positive regulation of reactive oxygen species metabolic process | 0.000105 | 0.002255 | 0.001825 |
| BP | GO:0002573 | myeloid leukocyte differentiation | 0.000106 | 0.002258 | 0.001827 |
| BP | GO:0001915 | negative regulation of T cell mediated cytotoxicity | 0.000108 | 0.00228 | 0.001845 |
| BP | GO:0035747 | natural killer cell chemotaxis | 0.000108 | 0.00228 | 0.001845 |
| BP | GO:0002710 | negative regulation of T cell mediated immunity | 0.000123 | 0.002579 | 0.002087 |
| BP | GO:0032635 | interleukin-6 production | 0.00013 | 0.002697 | 0.002183 |
| BP | GO:0032675 | regulation of interleukin-6 production | 0.00013 | 0.002697 | 0.002183 |
| BP | GO:0035743 | CD4-positive, alpha-beta T cell cytokine production | 0.000136 | 0.002822 | 0.002284 |
| BP | GO:0042060 | wound healing | 0.000144 | 0.002959 | 0.002395 |
| BP | GO:0046651 | lymphocyte proliferation | 0.000146 | 0.002963 | 0.002398 |
| BP | GO:0048708 | astrocyte differentiation | 0.000147 | 0.002963 | 0.002398 |
| BP | GO:0038093 | Fc receptor signaling pathway | 0.000147 | 0.002963 | 0.002398 |
| BP | GO:0043370 | regulation of CD4-positive, alpha-beta T cell differentiation | 0.000147 | 0.002963 | 0.002398 |
| BP | GO:0050832 | defense response to fungus | 0.000147 | 0.002963 | 0.002398 |
| BP | GO:0010575 | positive regulation of vascular endothelial growth factor production | 0.00015 | 0.003 | 0.002428 |
| BP | GO:0042130 | negative regulation of T cell proliferation | 0.000165 | 0.003276 | 0.002651 |
| BP | GO:0045622 | regulation of T-helper cell differentiation | 0.000166 | 0.003276 | 0.002651 |
| BP | GO:0097278 | complement-dependent cytotoxicity | 0.000166 | 0.003276 | 0.002651 |
| BP | GO:0002532 | production of molecular mediator involved in inflammatory response | 0.000176 | 0.003438 | 0.002782 |
| BP | GO:0002824 | positive regulation of adaptive immune response based on somatic recombination of immune receptors built from immunoglobulin superfamily domains | 0.000176 | 0.003438 | 0.002782 |
| BP | GO:0045953 | negative regulation of natural killer cell mediated cytotoxicity | 0.000178 | 0.003457 | 0.002798 |
| BP | GO:0034755 | iron ion transmembrane transport | 0.000182 | 0.003517 | 0.002846 |
| BP | GO:0006809 | nitric oxide biosynthetic process | 0.000183 | 0.003535 | 0.002861 |
| BP | GO:0031663 | lipopolysaccharide-mediated signaling pathway | 0.000189 | 0.003619 | 0.002929 |
| BP | GO:0045428 | regulation of nitric oxide biosynthetic process | 0.000189 | 0.003619 | 0.002929 |
| BP | GO:0006672 | ceramide metabolic process | 0.000191 | 0.003628 | 0.002936 |
| BP | GO:0016064 | immunoglobulin mediated immune response | 0.000204 | 0.003856 | 0.00312 |
| BP | GO:0002688 | regulation of leukocyte chemotaxis | 0.000205 | 0.003856 | 0.00312 |
| BP | GO:0045766 | positive regulation of angiogenesis | 0.000211 | 0.003946 | 0.003194 |
| BP | GO:1904018 | positive regulation of vasculature development | 0.000211 | 0.003946 | 0.003194 |
| BP | GO:0045055 | regulated exocytosis | 0.000214 | 0.003961 | 0.003206 |
| BP | GO:0002886 | regulation of myeloid leukocyte mediated immunity | 0.000214 | 0.003961 | 0.003206 |
| BP | GO:0062197 | cellular response to chemical stress | 0.000227 | 0.004148 | 0.003356 |
| BP | GO:0000272 | polysaccharide catabolic process | 0.000228 | 0.004148 | 0.003356 |
| BP | GO:0002716 | negative regulation of natural killer cell mediated immunity | 0.000228 | 0.004148 | 0.003356 |
| BP | GO:0050765 | negative regulation of phagocytosis | 0.000228 | 0.004148 | 0.003356 |
| BP | GO:0031623 | receptor internalization | 0.000237 | 0.004285 | 0.003468 |
| BP | GO:0019724 | B cell mediated immunity | 0.000241 | 0.004285 | 0.003468 |
| BP | GO:0001913 | T cell mediated cytotoxicity | 0.000241 | 0.004285 | 0.003468 |
| BP | GO:0080164 | regulation of nitric oxide metabolic process | 0.000241 | 0.004285 | 0.003468 |
| BP | GO:0009174 | pyrimidine ribonucleoside monophosphate biosynthetic process | 0.000244 | 0.004285 | 0.003468 |
| BP | GO:0043312 | neutrophil degranulation | 0.000244 | 0.004285 | 0.003468 |
| BP | GO:0045628 | regulation of T-helper 2 cell differentiation | 0.000244 | 0.004285 | 0.003468 |
| BP | GO:0106014 | regulation of inflammatory response to wounding | 0.000244 | 0.004285 | 0.003468 |
| BP | GO:0007229 | integrin-mediated signaling pathway | 0.000264 | 0.004583 | 0.003709 |
| BP | GO:0032611 | interleukin-1 beta production | 0.000264 | 0.004583 | 0.003709 |
| BP | GO:0032651 | regulation of interleukin-1 beta production | 0.000264 | 0.004583 | 0.003709 |
| BP | GO:0031960 | response to corticosteroid | 0.00027 | 0.004673 | 0.003782 |
| BP | GO:0009129 | pyrimidine nucleoside monophosphate metabolic process | 0.000288 | 0.004947 | 0.004004 |
| BP | GO:0030449 | regulation of complement activation | 0.000288 | 0.004947 | 0.004004 |
| BP | GO:0002701 | negative regulation of production of molecular mediator of immune response | 0.000294 | 0.004998 | 0.004045 |
| BP | GO:0043300 | regulation of leukocyte degranulation | 0.000294 | 0.004998 | 0.004045 |
| BP | GO:0032731 | positive regulation of interleukin-1 beta production | 0.000303 | 0.005122 | 0.004145 |
| BP | GO:0043030 | regulation of macrophage activation | 0.000303 | 0.005122 | 0.004145 |
| BP | GO:0002828 | regulation of type 2 immune response | 0.000309 | 0.005199 | 0.004207 |
| BP | GO:0045861 | negative regulation of proteolysis | 0.000321 | 0.005382 | 0.004356 |
| BP | GO:0045765 | regulation of angiogenesis | 0.000324 | 0.005412 | 0.004379 |
| BP | GO:0070664 | negative regulation of leukocyte proliferation | 0.000333 | 0.005528 | 0.004473 |
| BP | GO:0006898 | receptor-mediated endocytosis | 0.000337 | 0.005571 | 0.004509 |
| BP | GO:0045916 | negative regulation of complement activation | 0.000344 | 0.005676 | 0.004593 |
| BP | GO:0002719 | negative regulation of cytokine production involved in immune response | 0.000364 | 0.005986 | 0.004844 |
| BP | GO:0050670 | regulation of lymphocyte proliferation | 0.000372 | 0.006091 | 0.004929 |
| BP | GO:0019882 | antigen processing and presentation | 0.000387 | 0.006308 | 0.005105 |
| BP | GO:0048545 | response to steroid hormone | 0.000389 | 0.006308 | 0.005105 |
| BP | GO:1901342 | regulation of vasculature development | 0.000394 | 0.006374 | 0.005158 |
| BP | GO:0030101 | natural killer cell activation | 0.000427 | 0.006874 | 0.005563 |
| BP | GO:0032944 | regulation of mononuclear cell proliferation | 0.000451 | 0.007238 | 0.005857 |
| BP | GO:0034116 | positive regulation of heterotypic cell-cell adhesion | 0.000471 | 0.007509 | 0.006076 |
| BP | GO:0098883 | synapse pruning | 0.000471 | 0.007509 | 0.006076 |
| BP | GO:0070663 | regulation of leukocyte proliferation | 0.000507 | 0.008043 | 0.006508 |
| BP | GO:0009620 | response to fungus | 0.000519 | 0.008198 | 0.006634 |
| BP | GO:0048143 | astrocyte activation | 0.000543 | 0.008553 | 0.006922 |
| BP | GO:0032868 | response to insulin | 0.000553 | 0.008684 | 0.007028 |
| BP | GO:0046513 | ceramide biosynthetic process | 0.000574 | 0.008965 | 0.007255 |
| BP | GO:0002369 | T cell cytokine production | 0.000578 | 0.008965 | 0.007255 |
| BP | GO:0002724 | regulation of T cell cytokine production | 0.000578 | 0.008965 | 0.007255 |
| BP | GO:0061515 | myeloid cell development | 0.00058 | 0.008969 | 0.007258 |
| BP | GO:0050878 | regulation of body fluid levels | 0.000595 | 0.009169 | 0.00742 |
| BP | GO:0001659 | temperature homeostasis | 0.000611 | 0.009377 | 0.007588 |
| BP | GO:0002829 | negative regulation of type 2 immune response | 0.000629 | 0.009581 | 0.007754 |
| BP | GO:1902563 | regulation of neutrophil activation | 0.000629 | 0.009581 | 0.007754 |
| BP | GO:0045576 | mast cell activation | 0.000633 | 0.009617 | 0.007783 |
| BP | GO:0034341 | response to type II interferon | 0.000653 | 0.009871 | 0.007988 |
| BP | GO:0098581 | detection of external biotic stimulus | 0.000657 | 0.009871 | 0.007988 |
| BP | GO:1901623 | regulation of lymphocyte chemotaxis | 0.000657 | 0.009871 | 0.007988 |
| BP | GO:0002385 | mucosal immune response | 0.000667 | 0.009983 | 0.008079 |
| BP | GO:0071675 | regulation of mononuclear cell migration | 0.000731 | 0.010899 | 0.00882 |
| BP | GO:0032760 | positive regulation of tumor necrosis factor production | 0.000734 | 0.010914 | 0.008832 |
| BP | GO:0050672 | negative regulation of lymphocyte proliferation | 0.000748 | 0.011081 | 0.008967 |
| BP | GO:0033028 | myeloid cell apoptotic process | 0.000766 | 0.011255 | 0.009108 |
| BP | GO:0045730 | respiratory burst | 0.000766 | 0.011255 | 0.009108 |
| BP | GO:0002548 | monocyte chemotaxis | 0.000768 | 0.011255 | 0.009108 |
| BP | GO:0150146 | cell junction disassembly | 0.000788 | 0.011513 | 0.009317 |
| BP | GO:0032945 | negative regulation of mononuclear cell proliferation | 0.000812 | 0.011655 | 0.009432 |
| BP | GO:0034599 | cellular response to oxidative stress | 0.000814 | 0.011655 | 0.009432 |
| BP | GO:0009130 | pyrimidine nucleoside monophosphate biosynthetic process | 0.00082 | 0.011655 | 0.009432 |
| BP | GO:0009173 | pyrimidine ribonucleoside monophosphate metabolic process | 0.00082 | 0.011655 | 0.009432 |
| BP | GO:0016045 | detection of bacterium | 0.00082 | 0.011655 | 0.009432 |
| BP | GO:0032930 | positive regulation of superoxide anion generation | 0.00082 | 0.011655 | 0.009432 |
| BP | GO:0045019 | negative regulation of nitric oxide biosynthetic process | 0.00082 | 0.011655 | 0.009432 |
| BP | GO:1904406 | negative regulation of nitric oxide metabolic process | 0.00082 | 0.011655 | 0.009432 |
| BP | GO:0051384 | response to glucocorticoid | 0.000834 | 0.011816 | 0.009562 |
| BP | GO:0042554 | superoxide anion generation | 0.000877 | 0.012382 | 0.01002 |
| BP | GO:0045619 | regulation of lymphocyte differentiation | 0.00089 | 0.012529 | 0.010139 |
| BP | GO:0032732 | positive regulation of interleukin-1 production | 0.000925 | 0.012943 | 0.010474 |
| BP | GO:0006959 | humoral immune response | 0.000926 | 0.012943 | 0.010474 |
| BP | GO:0002675 | positive regulation of acute inflammatory response | 0.000938 | 0.012981 | 0.010504 |
| BP | GO:0031664 | regulation of lipopolysaccharide-mediated signaling pathway | 0.000938 | 0.012981 | 0.010504 |
| BP | GO:0090594 | inflammatory response to wounding | 0.000938 | 0.012981 | 0.010504 |
| BP | GO:0045824 | negative regulation of innate immune response | 0.000954 | 0.013159 | 0.010649 |
| BP | GO:1903557 | positive regulation of tumor necrosis factor superfamily cytokine production | 0.00098 | 0.013473 | 0.010903 |
| BP | GO:0002832 | negative regulation of response to biotic stimulus | 0.000996 | 0.013567 | 0.010979 |
| BP | GO:0120161 | regulation of cold-induced thermogenesis | 0.000996 | 0.013567 | 0.010979 |
| BP | GO:0032733 | positive regulation of interleukin-10 production | 0.001 | 0.013567 | 0.010979 |
| BP | GO:0140467 | integrated stress response signaling | 0.001 | 0.013567 | 0.010979 |
| BP | GO:0046637 | regulation of alpha-beta T cell differentiation | 0.001012 | 0.013691 | 0.011079 |
| BP | GO:0031640 | killing of cells of another organism | 0.001032 | 0.013872 | 0.011226 |
| BP | GO:0141061 | disruption of cell in another organism | 0.001032 | 0.013872 | 0.011226 |
| BP | GO:0045785 | positive regulation of cell adhesion | 0.001037 | 0.0139 | 0.011249 |
| BP | GO:0002921 | negative regulation of humoral immune response | 0.001049 | 0.013968 | 0.011303 |
| BP | GO:0006677 | glycosylceramide metabolic process | 0.001049 | 0.013968 | 0.011303 |
| BP | GO:0106106 | cold-induced thermogenesis | 0.001056 | 0.014013 | 0.01134 |
| BP | GO:0036037 | CD8-positive, alpha-beta T cell activation | 0.001108 | 0.014658 | 0.011862 |
| BP | GO:0002251 | organ or tissue specific immune response | 0.001135 | 0.014877 | 0.012039 |
| BP | GO:0032814 | regulation of natural killer cell activation | 0.001135 | 0.014877 | 0.012039 |
| BP | GO:0033003 | regulation of mast cell activation | 0.001135 | 0.014877 | 0.012039 |
| BP | GO:0006826 | iron ion transport | 0.001197 | 0.015638 | 0.012654 |
| BP | GO:0009615 | response to virus | 0.001217 | 0.015848 | 0.012825 |
| BP | GO:0034614 | cellular response to reactive oxygen species | 0.001252 | 0.01626 | 0.013158 |
| BP | GO:0032689 | negative regulation of type II interferon production | 0.001284 | 0.01657 | 0.013409 |
| BP | GO:2000516 | positive regulation of CD4-positive, alpha-beta T cell activation | 0.001284 | 0.01657 | 0.013409 |
| BP | GO:0043032 | positive regulation of macrophage activation | 0.001299 | 0.016721 | 0.013531 |
| BP | GO:0002440 | production of molecular mediator of immune response | 0.001313 | 0.016841 | 0.013629 |
| BP | GO:0090322 | regulation of superoxide metabolic process | 0.001514 | 0.019369 | 0.015674 |
| BP | GO:1902622 | regulation of neutrophil migration | 0.001626 | 0.020476 | 0.01657 |
| BP | GO:0002544 | chronic inflammatory response | 0.001635 | 0.020476 | 0.01657 |
| BP | GO:0009251 | glucan catabolic process | 0.001635 | 0.020476 | 0.01657 |
| BP | GO:0032695 | negative regulation of interleukin-12 production | 0.001635 | 0.020476 | 0.01657 |
| BP | GO:0032928 | regulation of superoxide anion generation | 0.001635 | 0.020476 | 0.01657 |
| BP | GO:0045064 | T-helper 2 cell differentiation | 0.001635 | 0.020476 | 0.01657 |
| BP | GO:0098543 | detection of other organism | 0.001635 | 0.020476 | 0.01657 |
| BP | GO:0045580 | regulation of T cell differentiation | 0.001678 | 0.020912 | 0.016923 |
| BP | GO:0002437 | inflammatory response to antigenic stimulus | 0.001684 | 0.020912 | 0.016923 |
| BP | GO:1903556 | negative regulation of tumor necrosis factor superfamily cytokine production | 0.001684 | 0.020912 | 0.016923 |
| BP | GO:0141060 | disruption of anatomical structure in another organism | 0.001734 | 0.021467 | 0.017371 |
| BP | GO:0002431 | Fc receptor mediated stimulatory signaling pathway | 0.001754 | 0.021467 | 0.017371 |
| BP | GO:0009218 | pyrimidine ribonucleotide metabolic process | 0.001754 | 0.021467 | 0.017371 |
| BP | GO:0070498 | interleukin-1-mediated signaling pathway | 0.001754 | 0.021467 | 0.017371 |
| BP | GO:0010573 | vascular endothelial growth factor production | 0.001765 | 0.021467 | 0.017371 |
| BP | GO:0032623 | interleukin-2 production | 0.001765 | 0.021467 | 0.017371 |
| BP | GO:0032663 | regulation of interleukin-2 production | 0.001765 | 0.021467 | 0.017371 |
| BP | GO:0072538 | T-helper 17 type immune response | 0.001821 | 0.022082 | 0.01787 |
| BP | GO:0008360 | regulation of cell shape | 0.001844 | 0.022305 | 0.01805 |
| BP | GO:0000041 | transition metal ion transport | 0.00186 | 0.022432 | 0.018153 |
| BP | GO:0071346 | cellular response to type II interferon | 0.001898 | 0.022822 | 0.018468 |
| BP | GO:0002712 | regulation of B cell mediated immunity | 0.001935 | 0.023072 | 0.018671 |
| BP | GO:0002889 | regulation of immunoglobulin mediated immune response | 0.001935 | 0.023072 | 0.018671 |
| BP | GO:0048247 | lymphocyte chemotaxis | 0.001935 | 0.023072 | 0.018671 |
| BP | GO:0120162 | positive regulation of cold-induced thermogenesis | 0.001993 | 0.023565 | 0.01907 |
| BP | GO:0002577 | regulation of antigen processing and presentation | 0.001999 | 0.023565 | 0.01907 |
| BP | GO:0071786 | endoplasmic reticulum tubular network organization | 0.001999 | 0.023565 | 0.01907 |
| BP | GO:0002685 | regulation of leukocyte migration | 0.002002 | 0.023565 | 0.01907 |
| BP | GO:0033032 | regulation of myeloid cell apoptotic process | 0.002021 | 0.023565 | 0.01907 |
| BP | GO:0036336 | dendritic cell migration | 0.002021 | 0.023565 | 0.01907 |
| BP | GO:0050869 | negative regulation of B cell activation | 0.002021 | 0.023565 | 0.01907 |
| BP | GO:0072539 | T-helper 17 cell differentiation | 0.002021 | 0.023565 | 0.01907 |
| BP | GO:0016052 | carbohydrate catabolic process | 0.00203 | 0.023568 | 0.019072 |
| BP | GO:1902107 | positive regulation of leukocyte differentiation | 0.002038 | 0.023568 | 0.019072 |
| BP | GO:1903708 | positive regulation of hemopoiesis | 0.002038 | 0.023568 | 0.019072 |
| BP | GO:0032613 | interleukin-10 production | 0.002118 | 0.024356 | 0.01971 |
| BP | GO:0032653 | regulation of interleukin-10 production | 0.002118 | 0.024356 | 0.01971 |
| BP | GO:0021782 | glial cell development | 0.002146 | 0.024612 | 0.019917 |
| BP | GO:0050920 | regulation of chemotaxis | 0.002177 | 0.024908 | 0.020156 |
| BP | GO:0033034 | positive regulation of myeloid cell apoptotic process | 0.002208 | 0.025058 | 0.020278 |
| BP | GO:0070391 | response to lipoteichoic acid | 0.002208 | 0.025058 | 0.020278 |
| BP | GO:0071223 | cellular response to lipoteichoic acid | 0.002208 | 0.025058 | 0.020278 |
| BP | GO:0090022 | regulation of neutrophil chemotaxis | 0.002316 | 0.02621 | 0.02121 |
| BP | GO:0070665 | positive regulation of leukocyte proliferation | 0.002363 | 0.02667 | 0.021582 |
| BP | GO:0046514 | ceramide catabolic process | 0.002416 | 0.026905 | 0.021773 |
| BP | GO:0090026 | positive regulation of monocyte chemotaxis | 0.002416 | 0.026905 | 0.021773 |
| BP | GO:1901739 | regulation of myoblast fusion | 0.002416 | 0.026905 | 0.021773 |
| BP | GO:2001044 | regulation of integrin-mediated signaling pathway | 0.002416 | 0.026905 | 0.021773 |
| BP | GO:2001185 | regulation of CD8-positive, alpha-beta T cell activation | 0.002416 | 0.026905 | 0.021773 |
| BP | GO:0050671 | positive regulation of lymphocyte proliferation | 0.002433 | 0.027009 | 0.021857 |
| BP | GO:0032642 | regulation of chemokine production | 0.002438 | 0.027009 | 0.021857 |
| BP | GO:0030041 | actin filament polymerization | 0.002484 | 0.027444 | 0.022209 |
| BP | GO:0034612 | response to tumor necrosis factor | 0.002496 | 0.02751 | 0.022262 |
| BP | GO:0001914 | regulation of T cell mediated cytotoxicity | 0.002512 | 0.027516 | 0.022267 |
| BP | GO:0006693 | prostaglandin metabolic process | 0.002512 | 0.027516 | 0.022267 |
| BP | GO:0006956 | complement activation | 0.002523 | 0.027516 | 0.022267 |
| BP | GO:0070301 | cellular response to hydrogen peroxide | 0.002523 | 0.027516 | 0.022267 |
| BP | GO:0017157 | regulation of exocytosis | 0.002575 | 0.028011 | 0.022668 |
| BP | GO:0045637 | regulation of myeloid cell differentiation | 0.002596 | 0.028096 | 0.022736 |
| BP | GO:0032602 | chemokine production | 0.002603 | 0.028096 | 0.022736 |
| BP | GO:0042102 | positive regulation of T cell proliferation | 0.002603 | 0.028096 | 0.022736 |
| BP | GO:1990845 | adaptive thermogenesis | 0.002609 | 0.028097 | 0.022737 |
| BP | GO:0051258 | protein polymerization | 0.002776 | 0.029802 | 0.024117 |
| BP | GO:0006692 | prostanoid metabolic process | 0.002782 | 0.029802 | 0.024117 |
| BP | GO:0032946 | positive regulation of mononuclear cell proliferation | 0.002854 | 0.030493 | 0.024676 |
| BP | GO:0010952 | positive regulation of peptidase activity | 0.002876 | 0.030632 | 0.024788 |
| BP | GO:1903305 | regulation of regulated secretory pathway | 0.002881 | 0.030632 | 0.024788 |
| BP | GO:0002765 | immune response-inhibiting signal transduction | 0.002974 | 0.031111 | 0.025176 |
| BP | GO:0006222 | UMP biosynthetic process | 0.002974 | 0.031111 | 0.025176 |
| BP | GO:0019372 | lipoxygenase pathway | 0.002974 | 0.031111 | 0.025176 |
| BP | GO:0045625 | regulation of T-helper 1 cell differentiation | 0.002974 | 0.031111 | 0.025176 |
| BP | GO:0070669 | response to interleukin-2 | 0.002974 | 0.031111 | 0.025176 |
| BP | GO:0071492 | cellular response to UV-A | 0.002974 | 0.031111 | 0.025176 |
| BP | GO:0002711 | positive regulation of T cell mediated immunity | 0.002985 | 0.031111 | 0.025176 |
| BP | GO:0045670 | regulation of osteoclast differentiation | 0.002985 | 0.031111 | 0.025176 |
| BP | GO:0070670 | response to interleukin-4 | 0.002999 | 0.031175 | 0.025228 |
| BP | GO:0032692 | negative regulation of interleukin-1 production | 0.003072 | 0.031863 | 0.025785 |
| BP | GO:0051260 | protein homooligomerization | 0.003083 | 0.031897 | 0.025812 |
| BP | GO:2000116 | regulation of cysteine-type endopeptidase activity | 0.003222 | 0.033255 | 0.026911 |
| BP | GO:0002752 | cell surface pattern recognition receptor signaling pathway | 0.003239 | 0.033348 | 0.026986 |
| BP | GO:1903034 | regulation of response to wounding | 0.003317 | 0.034069 | 0.02757 |
| BP | GO:0008154 | actin polymerization or depolymerization | 0.003366 | 0.034217 | 0.02769 |
| BP | GO:0071622 | regulation of granulocyte chemotaxis | 0.003385 | 0.034217 | 0.02769 |
| BP | GO:0070269 | pyroptosis | 0.003389 | 0.034217 | 0.02769 |
| BP | GO:0045621 | positive regulation of lymphocyte differentiation | 0.003411 | 0.034217 | 0.02769 |
| BP | GO:0002223 | stimulatory C-type lectin receptor signaling pathway | 0.003421 | 0.034217 | 0.02769 |
| BP | GO:0046835 | carbohydrate phosphorylation | 0.003421 | 0.034217 | 0.02769 |
| BP | GO:0072574 | hepatocyte proliferation | 0.003421 | 0.034217 | 0.02769 |
| BP | GO:0072575 | epithelial cell proliferation involved in liver morphogenesis | 0.003421 | 0.034217 | 0.02769 |
| BP | GO:1990840 | response to lectin | 0.003421 | 0.034217 | 0.02769 |
| BP | GO:1990858 | cellular response to lectin | 0.003421 | 0.034217 | 0.02769 |
| BP | GO:2000193 | positive regulation of fatty acid transport | 0.003421 | 0.034217 | 0.02769 |
| BP | GO:0008637 | apoptotic mitochondrial changes | 0.003563 | 0.035466 | 0.0287 |
| BP | GO:0030148 | sphingolipid biosynthetic process | 0.003563 | 0.035466 | 0.0287 |
| BP | GO:0006220 | pyrimidine nucleotide metabolic process | 0.003721 | 0.036952 | 0.029903 |
| BP | GO:0070098 | chemokine-mediated signaling pathway | 0.003793 | 0.037268 | 0.030159 |
| BP | GO:0006801 | superoxide metabolic process | 0.003797 | 0.037268 | 0.030159 |
| BP | GO:0014823 | response to activity | 0.003797 | 0.037268 | 0.030159 |
| BP | GO:0032722 | positive regulation of chemokine production | 0.003797 | 0.037268 | 0.030159 |
| BP | GO:0046635 | positive regulation of alpha-beta T cell activation | 0.003797 | 0.037268 | 0.030159 |
| BP | GO:0046466 | membrane lipid catabolic process | 0.003815 | 0.037365 | 0.030237 |
| BP | GO:0002645 | positive regulation of tolerance induction | 0.003885 | 0.037693 | 0.030503 |
| BP | GO:0010944 | negative regulation of transcription by competitive promoter binding | 0.003885 | 0.037693 | 0.030503 |
| BP | GO:0070672 | response to interleukin-15 | 0.003885 | 0.037693 | 0.030503 |
| BP | GO:1901503 | ether biosynthetic process | 0.003885 | 0.037693 | 0.030503 |
| BP | GO:0022604 | regulation of cell morphogenesis | 0.003937 | 0.038118 | 0.030846 |
| BP | GO:0071248 | cellular response to metal ion | 0.003996 | 0.038267 | 0.030967 |
| BP | GO:0030168 | platelet activation | 0.004015 | 0.038267 | 0.030967 |
| BP | GO:0006925 | inflammatory cell apoptotic process | 0.004016 | 0.038267 | 0.030967 |
| BP | GO:0009220 | pyrimidine ribonucleotide biosynthetic process | 0.004016 | 0.038267 | 0.030967 |
| BP | GO:0034114 | regulation of heterotypic cell-cell adhesion | 0.004016 | 0.038267 | 0.030967 |
| BP | GO:0044090 | positive regulation of vacuole organization | 0.004016 | 0.038267 | 0.030967 |
| BP | GO:0051043 | regulation of membrane protein ectodomain proteolysis | 0.004016 | 0.038267 | 0.030967 |
| BP | GO:0045444 | fat cell differentiation | 0.004087 | 0.038856 | 0.031444 |
| BP | GO:0071677 | positive regulation of mononuclear cell migration | 0.004101 | 0.038901 | 0.03148 |
| BP | GO:0006639 | acylglycerol metabolic process | 0.004234 | 0.040074 | 0.032429 |
| BP | GO:0010950 | positive regulation of endopeptidase activity | 0.004267 | 0.040294 | 0.032607 |
| BP | GO:0097242 | amyloid-beta clearance | 0.004278 | 0.040309 | 0.03262 |
| BP | GO:2000106 | regulation of leukocyte apoptotic process | 0.004332 | 0.040726 | 0.032957 |
| BP | GO:0006638 | neutral lipid metabolic process | 0.004463 | 0.04179 | 0.033818 |
| BP | GO:0070542 | response to fatty acid | 0.004465 | 0.04179 | 0.033818 |
| BP | GO:0043302 | positive regulation of leukocyte degranulation | 0.004678 | 0.043586 | 0.035272 |
| BP | GO:0072576 | liver morphogenesis | 0.004678 | 0.043586 | 0.035272 |
| BP | GO:0045806 | negative regulation of endocytosis | 0.004766 | 0.044307 | 0.035855 |
| BP | GO:0032570 | response to progesterone | 0.00478 | 0.044343 | 0.035884 |
| BP | GO:0002448 | mast cell mediated immunity | 0.004876 | 0.045135 | 0.036525 |
| BP | GO:0000302 | response to reactive oxygen species | 0.004915 | 0.045155 | 0.036541 |
| BP | GO:0032306 | regulation of prostaglandin secretion | 0.004947 | 0.045155 | 0.036541 |
| BP | GO:0032308 | positive regulation of prostaglandin secretion | 0.004947 | 0.045155 | 0.036541 |
| BP | GO:0032494 | response to peptidoglycan | 0.004947 | 0.045155 | 0.036541 |
| BP | GO:0070486 | leukocyte aggregation | 0.004947 | 0.045155 | 0.036541 |
| BP | GO:2001198 | regulation of dendritic cell differentiation | 0.004947 | 0.045155 | 0.036541 |
| BP | GO:0071241 | cellular response to inorganic substance | 0.004953 | 0.045155 | 0.036541 |
| BP | GO:0045582 | positive regulation of T cell differentiation | 0.005059 | 0.046022 | 0.037243 |
| BP | GO:0002690 | positive regulation of leukocyte chemotaxis | 0.00525 | 0.047657 | 0.038566 |
| BP | GO:0150077 | regulation of neuroinflammatory response | 0.005322 | 0.048206 | 0.03901 |
| BP | GO:1905523 | positive regulation of macrophage migration | 0.005409 | 0.048887 | 0.039561 |
| BP | GO:0002637 | regulation of immunoglobulin production | 0.005508 | 0.049676 | 0.0402 |
| BP | GO:1901654 | response to ketone | 0.005542 | 0.049879 | 0.040364 |
| CC | GO:0070820 | tertiary granule | 2.18E-42 | 8.71E-40 | 7.58E-40 |
| CC | GO:0042581 | specific granule | 9.99E-37 | 1.99E-34 | 1.73E-34 |
| CC | GO:0030667 | secretory granule membrane | 1.20E-30 | 1.59E-28 | 1.39E-28 |
| CC | GO:0034774 | secretory granule lumen | 9.04E-24 | 7.57E-22 | 6.59E-22 |
| CC | GO:0101002 | ficolin-1-rich granule | 9.49E-24 | 7.57E-22 | 6.59E-22 |
| CC | GO:0060205 | cytoplasmic vesicle lumen | 1.39E-23 | 9.12E-22 | 7.94E-22 |
| CC | GO:0031983 | vesicle lumen | 1.60E-23 | 9.12E-22 | 7.94E-22 |
| CC | GO:0070821 | tertiary granule membrane | 1.37E-22 | 6.86E-21 | 5.97E-21 |
| CC | GO:0035580 | specific granule lumen | 1.34E-20 | 5.92E-19 | 5.15E-19 |
| CC | GO:1904724 | tertiary granule lumen | 7.02E-18 | 2.80E-16 | 2.44E-16 |
| CC | GO:0035579 | specific granule membrane | 1.72E-17 | 6.22E-16 | 5.42E-16 |
| CC | GO:0101003 | ficolin-1-rich granule membrane | 1.32E-15 | 4.39E-14 | 3.82E-14 |
| CC | GO:0009897 | external side of plasma membrane | 3.44E-11 | 1.06E-09 | 9.19E-10 |
| CC | GO:1904813 | ficolin-1-rich granule lumen | 1.47E-10 | 4.18E-09 | 3.64E-09 |
| CC | GO:0005774 | vacuolar membrane | 1.68E-08 | 4.48E-07 | 3.90E-07 |
| CC | GO:0005766 | primary lysosome | 4.49E-08 | 1.05E-06 | 9.17E-07 |
| CC | GO:0042582 | azurophil granule | 4.49E-08 | 1.05E-06 | 9.17E-07 |
| CC | GO:0035577 | azurophil granule membrane | 3.84E-07 | 8.50E-06 | 7.40E-06 |
| CC | GO:0005765 | lysosomal membrane | 5.10E-07 | 1.02E-05 | 8.86E-06 |
| CC | GO:0098852 | lytic vacuole membrane | 5.10E-07 | 1.02E-05 | 8.86E-06 |
| CC | GO:0045335 | phagocytic vesicle | 7.63E-06 | 0.000145 | 0.000126 |
| CC | GO:0044194 | cytolytic granule | 1.56E-05 | 0.000282 | 0.000246 |
| CC | GO:0030139 | endocytic vesicle | 3.12E-05 | 0.000541 | 0.000471 |
| CC | GO:0030133 | transport vesicle | 5.45E-05 | 0.000906 | 0.000789 |
| CC | GO:0042470 | melanosome | 0.000236 | 0.003629 | 0.003159 |
| CC | GO:0048770 | pigment granule | 0.000236 | 0.003629 | 0.003159 |
| CC | GO:0030670 | phagocytic vesicle membrane | 0.000314 | 0.004643 | 0.004042 |
| CC | GO:0070382 | exocytic vesicle | 0.000634 | 0.009039 | 0.007869 |
| CC | GO:0061702 | canonical inflammasome complex | 0.000966 | 0.013286 | 0.011567 |
| CC | GO:0005775 | vacuolar lumen | 0.00107 | 0.014231 | 0.012389 |
| CC | GO:0030666 | endocytic vesicle membrane | 0.001293 | 0.016643 | 0.014489 |
| CC | GO:0062023 | collagen-containing extracellular matrix | 0.001619 | 0.020185 | 0.017573 |
| CC | GO:0005811 | lipid droplet | 0.002101 | 0.024699 | 0.021502 |
| CC | GO:0008021 | synaptic vesicle | 0.002126 | 0.024699 | 0.021502 |
| CC | GO:0045121 | membrane raft | 0.002215 | 0.024699 | 0.021502 |
| CC | GO:0031093 | platelet alpha granule lumen | 0.002228 | 0.024699 | 0.021502 |
| CC | GO:0098857 | membrane microdomain | 0.002297 | 0.024767 | 0.021562 |
| CC | GO:0071682 | endocytic vesicle lumen | 0.003158 | 0.033098 | 0.028815 |
| CC | GO:0031091 | platelet alpha granule | 0.003318 | 0.033098 | 0.028815 |
| CC | GO:0035578 | azurophil granule lumen | 0.003318 | 0.033098 | 0.028815 |
| CC | GO:0001931 | uropod | 0.004648 | 0.045229 | 0.039376 |
| CC | GO:0043202 | lysosomal lumen | 0.005215 | 0.049547 | 0.043135 |
| MF | GO:0140375 | immune receptor activity | 2.17E-21 | 1.56E-18 | 1.42E-18 |
| MF | GO:0004896 | cytokine receptor activity | 2.03E-09 | 7.28E-07 | 6.63E-07 |
| MF | GO:0030246 | carbohydrate binding | 7.16E-09 | 1.71E-06 | 1.56E-06 |
| MF | GO:0038187 | pattern recognition receptor activity | 3.65E-07 | 6.54E-05 | 5.95E-05 |
| MF | GO:0032393 | MHC class I receptor activity | 2.28E-06 | 0.000327 | 0.000298 |
| MF | GO:0016798 | hydrolase activity, acting on glycosyl bonds | 5.96E-06 | 0.000712 | 0.000648 |
| MF | GO:0004875 | complement receptor activity | 1.33E-05 | 0.001362 | 0.00124 |
| MF | GO:0019955 | cytokine binding | 2.05E-05 | 0.001838 | 0.001673 |
| MF | GO:0061134 | peptidase regulator activity | 3.77E-05 | 0.003002 | 0.002732 |
| MF | GO:0019865 | immunoglobulin binding | 4.92E-05 | 0.003524 | 0.003208 |
| MF | GO:0003953 | NAD+ nucleosidase activity | 6.66E-05 | 0.003675 | 0.003345 |
| MF | GO:0050135 | NAD(P)+ nucleosidase activity | 6.66E-05 | 0.003675 | 0.003345 |
| MF | GO:0061809 | NAD+ nucleotidase, cyclic ADP-ribose generating | 6.66E-05 | 0.003675 | 0.003345 |
| MF | GO:0061135 | endopeptidase regulator activity | 9.23E-05 | 0.004729 | 0.004305 |
| MF | GO:0032396 | inhibitory MHC class I receptor activity | 0.000127 | 0.006048 | 0.005505 |
| MF | GO:0005536 | glucose binding | 0.000194 | 0.008706 | 0.007925 |
| MF | GO:0070008 | serine-type exopeptidase activity | 0.000285 | 0.012011 | 0.010932 |
| MF | GO:0004553 | hydrolase activity, hydrolyzing O-glycosyl compounds | 0.000354 | 0.01411 | 0.012844 |
| MF | GO:0016799 | hydrolase activity, hydrolyzing N-glycosyl compounds | 0.000715 | 0.026992 | 0.024569 |
| MF | GO:0004866 | endopeptidase inhibitor activity | 0.00123 | 0.044079 | 0.040122 |
